# Targeted delivery of celastrol and siRNA via macrophage biomimetic nanoparticles for doxorubicin-induced cardiomyopathy therapy

**DOI:** 10.1016/j.mtbio.2026.103779

**Published:** 2026-10-23

**Authors:** Qiyu Zhang, Tianyu Guo, Xuna Xue, Zhibo Zhang, Xinyan Liu, Qing Cai, Ning Wang, Bo Zhang, Jinyong Peng

**Affiliations:** a Institute of Integrative Medicine, Dalian Medical University, Western 9 Lvshunnan Road, Dalian, 116044, China; b College of Pharmacy, Dalian Medical University, Western 9 Lvshunnan Road, Dalian, 116044, China; c Department of Cardiology, First Affiliated Hospital of Dalian Medical University, Dalian, 116044, China; d College of Pharmacy, Hubei Shizhen Laboratory, Hubei University of Chinese Medicine, Wuhan, 430065, China

**Keywords:** Doxorubicin-induced cardiomyopathy, Biomimetic nanoparticle, Celastrol, ADAM17, Inflammation/ferroptosis

## Abstract

Doxorubicin-induced cardiomyopathy (DIC) is a severe complication following anthracycline chemotherapy, and its main pathogenesis is inflammatory responses and ferroptosis. This study constructed hyaluronic acid-based macrophage biomimetic nanoparticles (M@HCDA) to co-delivery of Celastrol (Cel) and siADAM17 for the treatment of DIC. This research revealed that Cel exerted an anti-ferroptosis effect by regulating GPX4 and SLC7A11, while siADAM17 inhibited TAK1-MAPK pathway activation by silencing ADAM17. Meanwhile, the synergistic effect of Cel and siADAM17 significantly alleviated oxidative stress, myocardial fibrosis, apoptosis, and mitochondrial dysfunction, thereby relieving myocardial injury caused by DOX-induced cardiotoxicity and providing effective early cardioprotection. *In vitro* and *in vivo* experiments indicated that M@HCDA exhibited good stability, biocompatibility, and targeting capability, showing the clinical application potential. The design and development of M@HCDA provide new experimental evidence and candidate strategy for treating DIC.

## Highlights

• Macrophage membrane biomimetic nanoparticles M@HCDA can target damaged myocardium and co-deliver siADAM17 and Celastrol.
• Treatment of M@HCDA *in vitro* and *in vivo* suppressed doxorubicin-induced mitochondrial injury and rescued cardiac function.
• M@HCDA improves doxorubicin-induced ferroptosis by targeting and delivering Celastrol to regulate GPX4 and SLC7A11.
• M@HCDA alleviates doxorubicin-induced inflammation by inhibiting the MAPK signaling through targeted delivery of siADAM17.

## Graphical abstract

**Figure:**
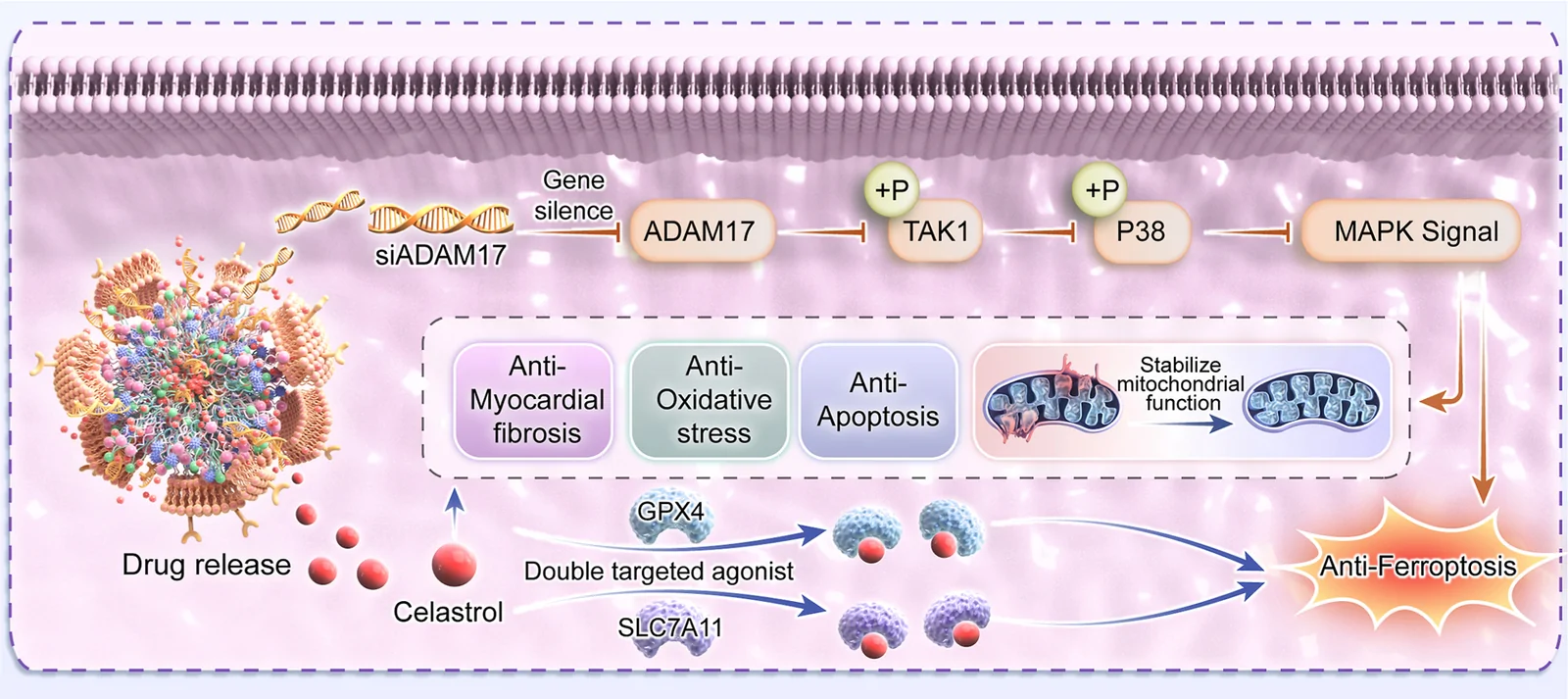

## 1 Introduction

Doxorubicin (DOX) is a prototypical anthracycline chemotherapy drug, exerts significant anti-tumor activity and is widely used for the treatment of various malignancies [1], [2], [3], [4], [5], [6]. Whereas, the clinical application of DOX is limited by its dose-dependent cardiotoxicity, leading to the occurrence of doxorubicin-induced cardiomyopathy (DIC), which manifests as irreversible and progressive myocardial injury and can lead to heart failure and cardiogenic shock in severe cases, posing a great threat to the lives of cancer patients [7], [8]. Currently, there is no standard therapeutic strategy for DIC, and Dexrazoxane is the only approved therapeutic option. It selectively chelates intracellular ferrous iron, thereby inhibiting Fenton reaction mediated ferroptosis. However, the true efficacy of Dexrazoxane in treating DIC remains controversial because of its significant side effects [9]. Therefore, developing a safe and effective therapeutic strategy for DIC is urgently needed.

To better understand the onset and progression of DIC, it is essential to elucidate its underlying pathological mechanisms. The early stages of DIC are characterized by inflammatory cell infiltration and aggregation. Various pathological changes occur as the disease progresses, including myocardial fibrosis, oxidative stress, mitochondrial dysfunction, and DNA damage [10], [11], [12], ultimately leading to myocardial cell death. Studies have shown that doxorubicin can directly induce apoptosis in cardiomyocytes via inflammatory factors [13]. Such data suggests that the inflammatory responses are critically involved in DIC development.

Furthermore, ferroptosis has emerged as another critical mechanism driving DIC progression. Ferroptosis is a form of iron-dependent programmed non-apoptotic cell death [14], driven by excessive intracellular Fe^2+^ that triggers the Fenton reaction, induces lipid peroxidation, and eventual cell death [15]. DOX can disrupt iron homeostasis by modulating intracellular iron-regulatory proteins, thereby further depleting GPX4 and glutathione (GSH) and promoting ferroptosis [16], thus accelerating the process of DIC. In addition, iron accumulation can activate inflammatory signaling pathways, produce pro-inflammatory factors, and exacerbate inflammation-induced myocardial injury [17]. Therefore, a dual-mode, synergistic therapeutic strategy that simultaneously targets inflammatory responses and ferroptosis holds promise for providing new insights into the prevention and treatment of DIC.

As a key mediator of inflammatory and apoptotic pathways in cardiovascular injury, A Disintegrin and Metalloproteinase 17 (ADAM17) has emerged as a promising therapeutic target for DIC. ADAM17 is an I-type transmembrane protease with a wide range of substrates, capable of mediating the shedding of extracellular domains of various membrane-bound factors, thereby activating multiple downstream signaling pathways [18]. ADAM17 plays a crucial role in various cardiovascular diseases, including myocardial infarction, diabetic cardiomyopathy and myocarditis [19], [20], [21], [22], [23]. A recent study has confirmed that ADAM17 can exacerbate DIC, and its mechanism may involve ADAM17 promoting TNF-α release, upregulating TRAF3 expression, enhancing TAK1 phosphorylation, and finally activating the MAPKs signaling pathway to induce cardiomyocyte apoptosis [24]. Moreover, in mice with cardiac-specific ADAM17 knockout, pathological manifestations, including cardiac dysfunction, myocardial fibrosis, and apoptosis, are significantly reduced. Small interfering RNA (siRNA), due to its high specificity in gene silencing, has been widely applied in basic and translational research. However, its *in vivo* application still faces bottlenecks, including low delivery efficiency, poor bioavailability, and a short circulation time. Therefore, developing siADAM17 nanomedicines that can be targeted and delivered to injured myocardium may become an important direction in the treatment of DIC.

Moreover, there remains a need for complementary cardioprotective agents, and natural products represent promising candidates. Celastrol (Cel), a pentacyclic triterpenoid natural product, exhibits various biological activities, including anti-inflammatory, antioxidant, antibacterial, and antitumor properties, and has attracted significant attention in recent years [25], [26], [27]. Among them, the effect of Cel against myocardial injury has attracted our particular attention. Many studies have shown that Cel have protective effect against cardiovascular diseases, including atherosclerosis, aortic valve disease, diabetic cardiomyopathy and heart failure [28], [29], [30], [31]. Previous studies have shown that Cel significantly inhibits Ang II-induced cardiac remodeling in mice [32] and myocardial fibrosis following myocardial infarction in rats [33], [34]. Moreover, intraperitoneal injection of Cel has also shown a certain degree of curative effects on subacute DIC [35]. However, its therapeutic effects in chronic DIC remain rarely reported. In addition, Cel faces challenges, including poor water solubility and low bioavailability, which severely limit its clinical translation.

Therefore, an efficient targeted delivery method is essential. Biomimetic nanoparticles (BNPs) have gradually emerged as a promising novel drug delivery platform [36]. Compared to traditional liposomes, BNPs exhibit significant advantages in homing targeting, biocompatibility, and immune evasion [37]. Among them, Macrophage membranes have been widely used in the construction of targeted nanomedicines for various diseases due to their relatively stable origin, high safety profile, and inherent ability to target inflammation [38], [39], [40]. Notably, studies have shown that macrophage membranes can target injured myocardial tissues, thereby enhancing the uptake of nanodrugs by myocardial cells under pathological conditions [41].

To further optimize this macrophage membrane-based delivery system, Hyaluronic acid (HA) was incorporated. HA is a non-toxic, biocompatible, highly hydrophilic, and non-immunogenic polyanionic glycosaminoglycan [42], widely used in the construction of nano-delivery systems. The current study designed and synthesized a HA-based macrophage membrane biomimetic nanoparticle, M@HCDA, for the co-delivery of Cel and siADAM17, aimed at treating DIC (Scheme 1). The mechanism by which M@HCDA exerts its anti-DIC effects is as follows: Firstly, leveraging the biological characteristics of macrophage membranes, M@HCDA can actively target damaged myocardial tissue and, upon cellular uptake, release siADAM17 and Cel. Next, siADAM17 silences ADAM17 expression in DIC myocardial tissue, thereby inhibiting the abnormal activation of the downstream TAK1-MAPK signaling pathway. Then, Cel targets and binds to GPX4 and SLC7A11 through hydrogen bonding and electrostatic interactions, thus exerting an anti-ferroptosis effect. Meanwhile, the synergistic action of both agents can further achieve multiple therapeutic effects, including anti-fibrosis, antioxidant, anti-apoptotic, and improvement of mitochondrial dysfunction. In summary, this study has developed a novel biomimetic nano-delivery system, which opens up new avenues for the targeted delivery of gene drugs, and provides new strategies for the prevention and treatment of DIC.

**Scheme 1:**
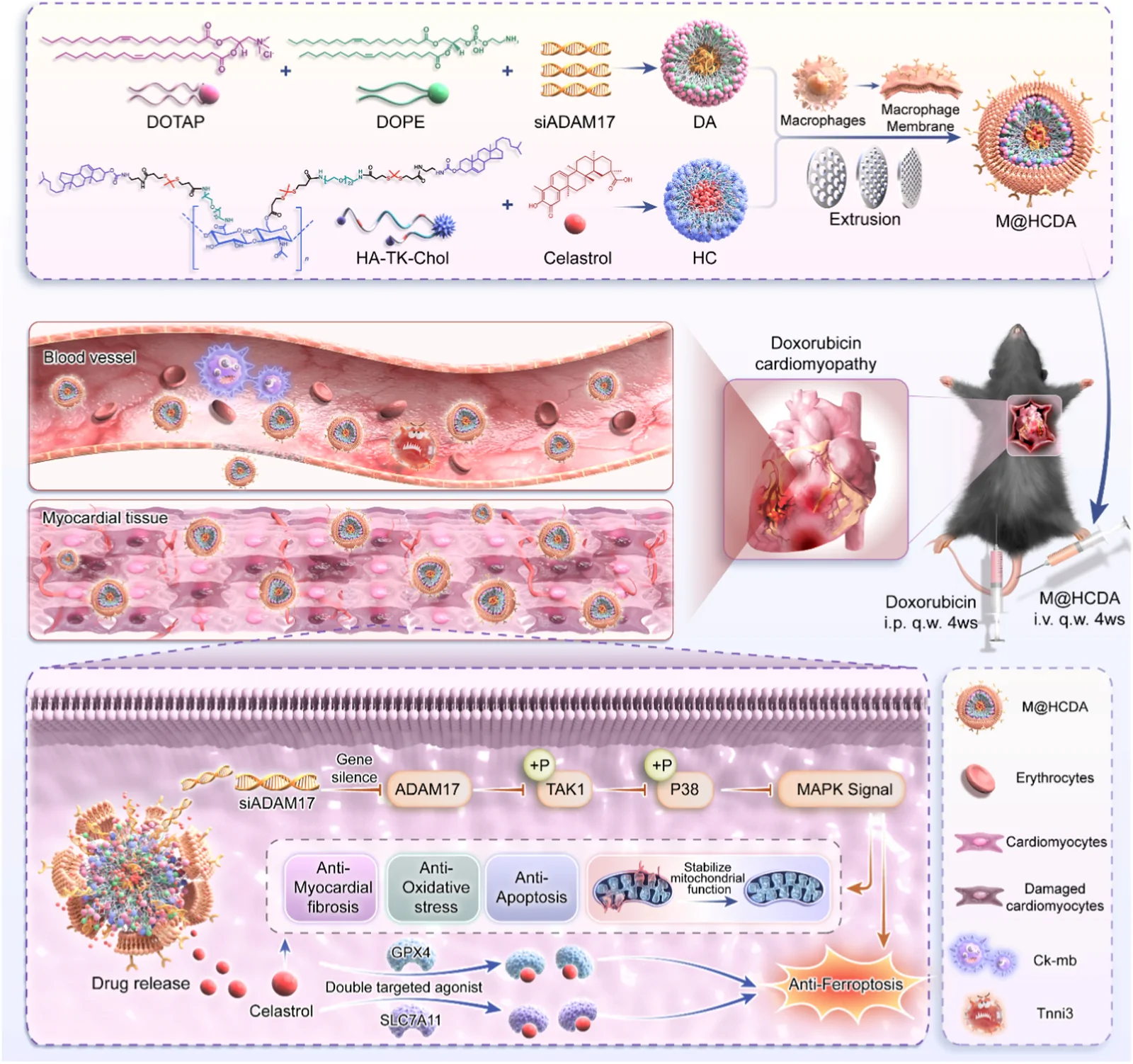
Structural composition of M@HCDA and its mechanism against doxorubicin cardiomyopathy.

## 2 Materials and methods

### 2.1 Cells and animals

The HL-1 atrial muscle cells were purchased from MeilunBio Co., Ltd. The AC16 human cardiomyocytes were purchased from Wuhan Procell Biotechnology Co., Ltd. The RAW 264.7 cells (Mouse monocyte-macrophage leukemia cell) were purchased from Wuhan SUNNCELL Biotechnology Co., Ltd. Male C57BL/6 mice (6 - 8 weeks) were purchased from Liaoning Changsheng Biotechnology Co., Ltd. All mice were housed in the controlled environment at 23 ± 2°C under a 12 h dark light cycle with free access to food and water. All animal experiments in this study followed the National Institutes of Health guidelines and were authorised by the Experimental Animal Welfare and Ethics Committee of Dalian Medical University (Ethics number: XL251128646).

### 2.2 Chemicals and materials

HA was purchased from Freda Biochem Co., LTD, China. (Weight-average molecular weight, Mw = 52,559 g mol^−1^; Number average molecular weight, Mn = 30,191 g mol^−1^; Mw/Mn = polydispersity = 1.74). Fluorescent dye DIR, N-(3-Dimethylaminopropyl)-N-ethylcarbodiimide (EDCI), Coumarin 6 (C6), 4-dimethylaminopyridine (DMAP), Cholesteryl chloroformate, DOTAP, DOPE, Polyoxyethylene bis-amine (NH_2_-PEG-NH_2_, 1k Da) and N-Hydroxysuccinimide (NHS) were purchased from Shanghai Macklin Biochemical Co., Ltd, China. 3-mercapto -propionic acid was supplied from Shanghai Aladdin biochemical technology co. LTD, China. Adriamycin hydrochloride (DOX·HCl) was purchased from Shanghai Hao Yun Chemical Technology Co., Ltd. Celastrol (HPLC ≥ 98%) was provided from NanJing Dilger Medical Technology Co., LTD, China. Ready-to-use DAPI staining solution and Cell Counting kit-8 (CCK-8) were purchased from Seven, Beijing, China. PMSF (IP02809) was purchased from Beijing Solarbio Science & Technology Co., Ltd. LDHA + LDHB mAb were purchased from HUABIO, Hangzhou, China. F4/80 mAb and TNNT2 mAb were purchased from ABclonal Biotechnology Co., Ltd. Lamin A/C Ab and p-P38 MAPK Ab were purchased from Affinity Biosciences. Phospho-TAK1 mAb and TAK1 mAb were purchased from Zen bioscience Limited.

### 2.3 Preparation of nanoparticles (NPs)

The thin-film hydration method was used to mix DOTAP (abbreviated as D) and DOPE (abbreviated as D) at a 1: 1 mass ratio, then dissolve them in chloroform (Table S1). Subsequently, the chloroform solution was evaporated under reduced pressure to form a uniform thin film, and then vacuum-dried. Next, ultrapure water was added to disperse the film until it became clear, thus preparing blank cationic lipid DD, which was stored at 4°C for later use. DD and siRNA (siADAM17) were mixed at different nitrogen-to-phosphorus (N/P) ratios, vortexed, and incubated at room temperature for 30 min to prepare siADAM17-loaded liposomes (DA).

In another operation, NPs encapsulating Cel were prepared via the nanoprecipitation method using HA-TK-PEG-Chol (H) and Cel (C) at a 3: 1 mass ratio. First, a certain amount of H was dissolved in dimethyl sulfoxide (DMSO), and then uniformly dispersed in a proper volume of PBS. Next, a specified amount of C was dissolved in a small volume DMSO, then added dropwise slowly and uniformly to the H solution. After stirring for 3-5 h at room temperature, dialysis was performed for 12 h using a 3500 Da dialysis bag in PBS to remove DMSO from the sample. Finally, the dialyzed solution was aspirated and centrifuged at 3000 rpm for 5 min, and the supernatant was collected as HC. Eventually, HC and DA were mixed at a 1: 1 mass ratio of Cel and siADAM17. After vortexed, the mixture was incubated at room temperature for 60 min to obtain NPs encapsulating Cel and siADAM17 (HCDA).

Isolate the monocyte-macrophage cell membrane according to the previously reported method [43], [44]. Briefly, Culture RAW 264.7 cells in medium containing 10% fetal bovine serum (FBS). Washing cells three times with PBS, then adding an appropriate volume of PBS containing EDTA and collect cells with a cell scraper. Centrifuge and collect the cell precipitate, lysed using a cell lysis buffer containing PMSF, and then perform freeze-thaw cycle for three times to further disrupt the cells. After centrifugation at 4°C and 700 × g for 10 min, the supernatant was collected for further use, while the precipitate consists of cell nuclei and unbroken cells. Then centrifugation at 4°C and 14,000 × g for 30 min, collecting the cytoplasm supernatant and the cell membrane sediment separately.

Add the macrophage cell membrane solution to the HCDA NPs at a membrane-to-phosphatidylcholine (PC) mass ratio of 1:1, and sonicate for 3 min to wrap the membrane around the NPs. Using a mini nanotech extruder, the above solution was repeatedly extruded through polycarbonate membranes with pore sizes of 1000 nm and then 200 nm in sequence for screening and separation. Finally, macrophage cell membrane-coated HCDA BNPs (M@HCDA) with diameters of about 100-200 nm were obtained for later use.

In order to confirm the successful preparation of M@HCDA, equal volumes of macrophage whole-cell lysate, HCDA solution, and M@HCDA solution were each mixed with loading buffer, and the gel electrophoresis experiment was carried out, followed by Coomassie Brilliant Blue staining.

Fluorescent NPs were prepared for *in vitro* cellular uptake experiment. The Cel and siADAM17 were replaced with C6 to obtain the targeted NPs M@HD@C6 and nontargeted NPs HD@C6. Furthermore, another fluorescent NPs were prepared for the *in vivo* imaging experiment. DIR was used to replace both to synthesize targeted NPs M@HD@DIR and nontargeted NPs HD@DIR. All other steps were performed as described above.

### 2.4 Statistical analysis

To reduce subjective bias in the experiment, we adopted a single-blind method for experimental operations and data statistics. All results are represented as mean ± SD. Significant difference was analyzed by GraphPad Prism 8.0. The t tests were performed to compare the differences between two groups. One-way ANOVA was performed to examine the differences among multiple groups. A value of *p* < 0.05 was considered statistically (*p < 0.05, **p < 0.01).

Remaining the methodologies were described in the supplementary information.

## 3 Results and discussion

### 3.1 Preparation and characterization of M@HCDA

To develop a BNP capable of co-delivering Cel and siRNA, this study first designed and synthesized a ROS-responsive amphiphilic molecule, HA-TK-PEG-Chol (H). The structure was systematically characterized using high-resolution mass spectrometry, NMR spectroscopy, diffusion-ordered spectroscopy (DOSY), and gel permeation chromatography (GPC). The results confirmed the successful synthesis of each intermediate and the intended product HA-TK-PEG-Chol (Supporting Information Fig. S1, S2).

HA-TK-PEG-Chol can self-assemble into nanostructures in PBS and further encapsulate Cel, yielding nanocarriers H@Cel (HC). Additionally, upon combining DOTAP and DOPE, a positively charged lipid complex, DD, is formed, which can bind to negatively charged siRNA via electrostatic interactions, thereby forming nanocarriers DA loaded with siADAM17. Then, HC is mixed with DA and an equal mass of macrophage membrane (M), and after sonication and co-extrusion, biomimetic nanocarriers M@HCDA are obtained, forming a drug/gene co-delivery system targeting damaged myocardial tissue (Scheme 1). The premise for constructing the siADAM17 delivery system is to determine the effective siRNA sequences targeting ADAM17. As shown in Supporting Information Fig. S3, among the two siRNA sequences targeting ADAM17 designed, only the expression of ADAM17 protein corresponding to siADAM17-1 was significantly down-regulated, demonstrating that the siADAM17-1 can effectively and stably silence ADAM17. Based on this, further optimization of the combining conditions between the nanocarrier and siADAM17-1 was performed using agarose gel electrophoresis, which indicated that the optimal N/P ratio of HCDD to siADAM17 is 4:1 (Supporting Information Fig. S4).

To prepare the drug/gene co-delivery BNPs M@HCDA, we first isolated and purified the macrophage membrane, as shown in Fig. 1A. To verify the accuracy of the isolated, proteins were extracted separately from the whole cells, nuclei, cytoplasm, and membrane groups, and characterized the specific markers F4/80 and CD68 of RAW264.7 macrophage membranes by Western blot. The results showed that both the whole-cell and membrane groups expressed F4/80 and CD68 (Fig. 1B). Meanwhile, the cytoplasmic markers LDHA and LDHB, as well as the nuclear marker Lamin A/C, were only detected enriched in their corresponding fractions, with no significant expression observed in the membrane group. Subsequently, HCDA was mixed with an equal mass of macrophage membrane, subjected to ultrasonic treatment and extruded, final yielding M@HCDA BNPs. The encapsulation efficiencies of celastrol in HCDA and M@HCDA were 90.1% and 77.0% respectively. The encapsulation efficiencies of siADAM17 in HCDA and M@HCDA were 74.6% and 72.3% respectively. The drug loading capacity of celastrol in HCDA and M@HCDA were 3% and 2.6% respectively. Dynamic light scattering (DLS) and transmission electron microscopy (TEM) were used to characterize the particle size, polydispersity index (PDI), and morphology of HCDA and M@HCDA, as well as the ζ potential of each component. As shown in Fig. 1C, the ζ potential of M@HCDA decreased to 7.4 ± 0.38 mV, while maintaining a unimodal and homogeneous distribution characteristic (Supporting Information Fig. S5). The physical images and DLS characterization results of HCDA and M@HCDA were shown in Fig. 1D and E, respectively. Compared to HCDA, M@HCDA exhibited a more pronounced halo-like refractive phenomenon, indicating a change in its surface structure. The DLS results revealed that the average particle size and PDI of HCDA were 175.47 ± 7.5 nm and 0.116 ± 0.037 respectively, and those of M@HCDA were 157.67 ± 4.04 nm and 0.12 ± 0.013 respectively, indicating that both NPs had favourable dispersibility and uniformity. Referring to TEM (Fig. 1F), HCDA exhibited a generally spherical structure, but the outer layer of M@HCDA particles showed a distinct and light-coloured membrane-like coating, and the interior consists of dark, dense particles, which confirmed the successful encapsulation of macrophage membranes on HCDA surface. The results of Coomassie Brilliant Blue staining showed that the protein band patterns of M@HCDA were similar to those of the macrophage membrane, while HCDA had no corresponding protein expression (Fig. 1G), further confirming the successful encapsulation of macrophage membranes on the NPs' surfaces. Based on the above experiments, it could be demonstrated that the BNPs M@HCDA have been successfully constructed.

**Fig. 1:**
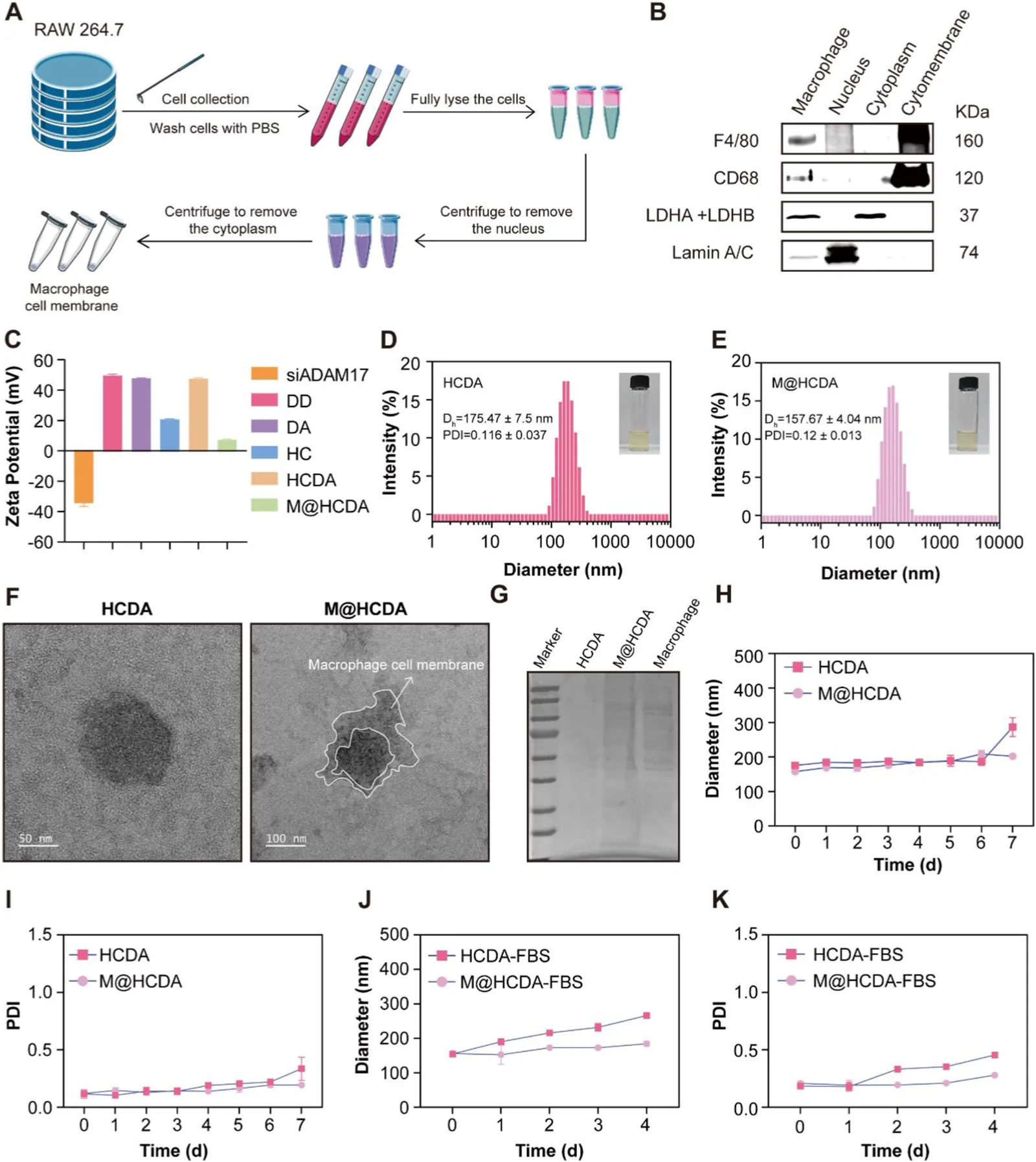
Characterization of M@HCDA. (A) Flow chart of RAW264.7 macrophage cell membrane isolation. (B) Immunoblotting images of F4/80, CD68, LDHA + LDHB and Lamin A/C in various components of macrophages. (C) Zeta potential of siADAM17, DD, DA, HC, HCDA and M@HCDA (n = 3). (D) The particle size distribution of HCDA (n = 3). (E) The particle size distribution of M@HCDA (n = 3). (F) TEM images of HCDA and M@HCDA. (G) SDS-PAGE image of HCDA, M@HCDA and Macrophage. Particle size (H) and PDI (I) of HCDA and M@HCDA within 7 days (n = 3). Particle size (J) and PDI (K) of HCDA and M@HCDA within 4 days in 10% FBS environment (n = 3).

Moreover, the storage stability, serum stability and nuclease stability of HCDA and M@HCDA were evaluated separately. The results of the storage stability experiment showed that M@HCDA had almost no significant changes in particle size and PDI compared to its initial state, particularly on day 7. In contrast, HCDA showed more pronounced fluctuations with poorer storage stability (Fig. 1H and I). In the serum stability experiment, the particle size and PDI of M@HCDA remained largely unchanged throughout the detection process. In contrast, those of HCDA gradually increased over time, suggesting a greater tendency toward aggregation or structural instability in serum environments (Fig. 1J and K). In the nuclease stability experiment, since siRNA cannot leave distinct imprints on the agarose gel after being encapsulated inside NPs, we choose to observed the imprints of the complexes formed by siRNA and proteins in FBS (such as serum proteins) to reflected the leakage of siRNA in a 10% FBS environment. In other words, the higher the amount of siRNA–protein complexes and the earlier they appear, the weaker the protective ability of NPs against siRNA. As seen from Supporting Information Fig. S6, naked siADAM17 was rapidly degraded almost immediately upon the addition of 10% FBS, and began to form a large number of complexes at 10 min. As time extends, the number of complexes gradually increased and reached a peak. However, the time points at which HCDA and M@HCDA formed a large number of complexes were both later than that for naked siADAM17, which were 15 min and 20 min respectively. These results indicate that M@HCDA possesses good storage stability, serum stability and nuclease stability. The superior stability of M@HCDA compared to HCDA may primarily be attributed to the biomimetic protective effect provided by the macrophage membrane coating. On one hand, the cell membrane forms a natural barrier on the NPs surfaces, effectively reducing the direct particle exposure to the external environment, thereby minimizing nonspecific interparticle interactions and aggregation. On the other hand, the membrane proteins and phospholipid bilayer on the cell membrane surface contribute to enhancing the interfacial stability of the NPs in physiological environments, prevent nuclease invasion, and mitigating serum protein adsorption and the aggregation induced by it. Furthermore, the membrane coating can buffer the disturbances of NPs core induced by external ionic strength and complex biological media, thereby enabling M@HCDA to possess both superior stability and serum stability.

### 3.2 Preventive effect of M@HCDA on Dox-induced cardiomyocyte injury

As illustrated in Fig. 2A, before validating the pharmacological activity of M@HCDA, it is essential to clarify its safety dosage and the DIC modelling conditions *in vitro*, and the preventive effects of M@HCDA and its related intermediate on the DIC model *in vitro*. Besides mouse HL-1 atrial muscle cells, this study also selected human AC16 myocardial cells, for related experiments to enhance the clinical translational value. As shown in Fig. 2B and C, in any of the cell types, there were no significant differences in viability compared to the 0 nM group. The Live/Dead cell staining results also indicated that none of the dosage groups caused significant cell death (Fig. 2D and E). These results suggest that M@HCDA exhibits good biocompatibility *in vitro*. Subsequently, the CCK-8 test was employed to determine the DIC modelling conditions *in vitro*. The results indicated that when the DOX concentration was 1 μM, the survival rate of both cells was about 50%, so 1 μM was selected as the DIC modelling dose (Fig. 2F and G). Based on this, the preventive effects of various drugs on the DIC *in vitro* model were further evaluated. As shown in Fig. 2H and I, compared to the DOX group, the Mac, HD, Cel, and M@DA groups exhibited weaker protective effects against DOX-induced cell damage. In contrast, HCDA, as a “dual-mode drug,” demonstrated a significantly greater improvement in cell viability than the “single-mode” formulation M@HC. More importantly, the protective effect of M@HCDA even exceeded that of HCDA, with a clear dose-dependent relationship. In short, M@HCDA can effectively reduce DOX-induced damage in HL-1 and AC16 cells, showing strong anti-DIC activity and safety *in vitro*.

**Fig. 2:**
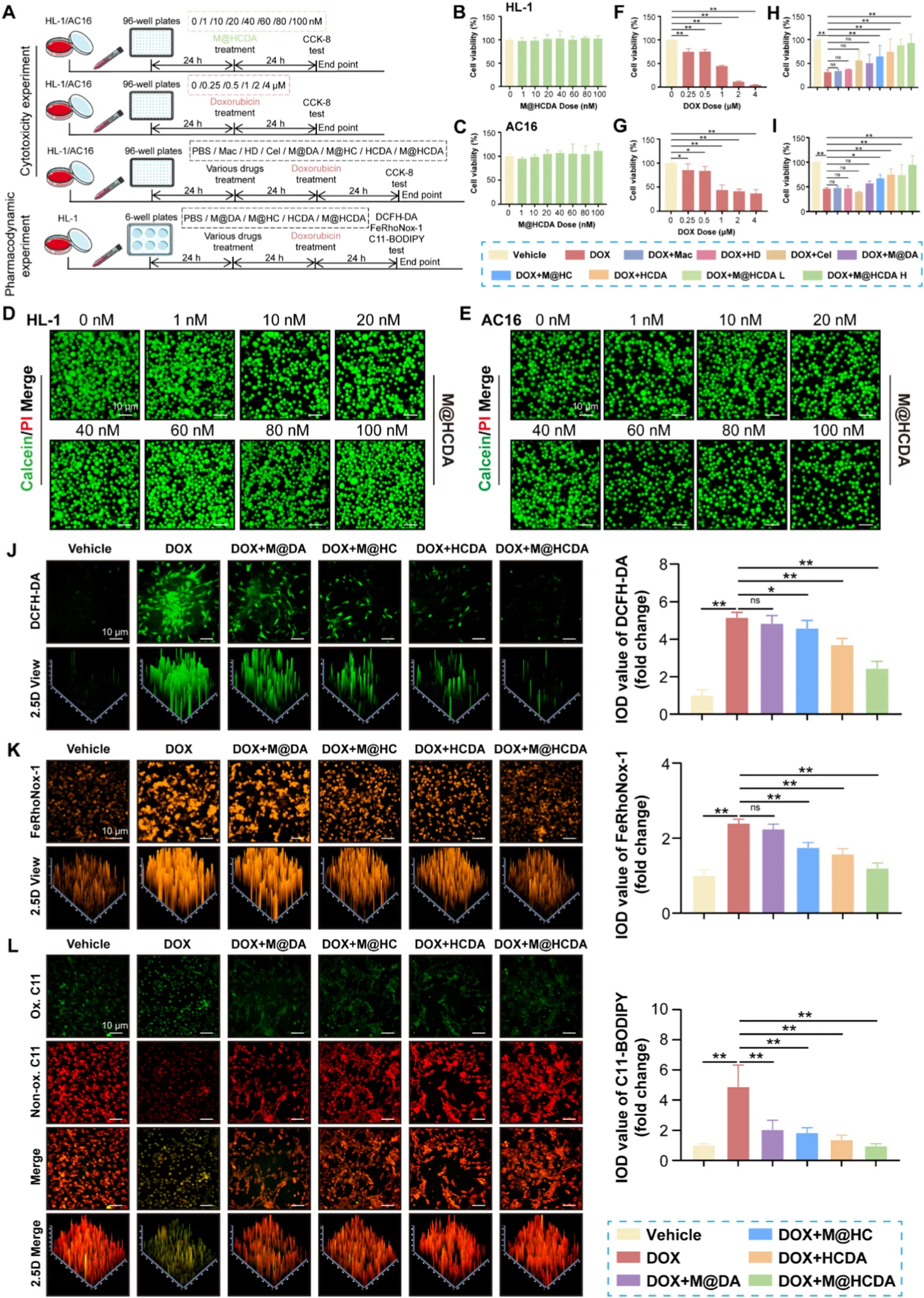
Preventive effect of M@HCDA on DIC *in vitro*. (A) Schematic diagram of experimental workflow for cytotoxicity and pharmacodynamics of various NPs. Cell viability levels of HL-1 cells (B) and AC16 cells (C) after 24 h treatment of M@HCDA. Live/Dead cell staining images of HL-1 cells (D) and AC16 cells (E) after 24 h treatment of M@HCDA. Cytotoxicity levels in HL-1 cells (F) and AC16 cells (G) after 24 h treatment of DOX. Cell viability levels after treatment of HL-1 cells (H) and AC16 cells (I) with different formulations for 24 h, followed by intervention with DOX for an additional 24 h. Fluorescence images of DCFH-DA staining and quantitative analysis (J), FeRhoNox-1 staining and quantitative analysis (K), C11-BODIPY staining and quantitative analysis (L) in HL-1 cells after treatment with different formulations. (n = 6). Scale bar, 10 μm. The data are presented as the mean ± SD; Statistical differences were determined by one-way ANOVA; *p < 0.05, **p < 0.01.

According to above results, six groups were selected for subsequent pharmacological experiments, namely Vehicle, DOX, DOX + M@DA, DOX + M@HC, DOX + HCDA, and DOX + M@HCDA (Fig. 2A). Considering that both oxidative stress and ferroptosis are important to DIC development, mitochondrial dysfunction and apoptosis, this study further evaluated the mechanism of M@HCDA with respect to ROS generation, Fe^2+^ accumulation, and lipid peroxidation. Corresponding to Fig. 2J and K, ROS levels and Fe^2+^ content in cells increased significantly after DOX treatment, indicating marked oxidative stress and iron ion accumulation. Compared to the DOX group, the ROS generation and Fe^2+^ accumulation of the DOX + M@HCDA group was significantly inhibited, and other treatment groups also showed varying degrees of alleviation, implying that M@HCDA possesses strong antioxidant stress and anti-iron ion accumulation capabilities. As presented in Fig. 2L, compared to the Vehicle group, the DOX group exhibited a significant fluorescence enhancement of oxidized C11-BODIPY (Ox. C11) and a significant fluorescence attenuation of reduced C11-BODIPY (Non-ox. C11), which revealed a substantial occurrence of lipid peroxidation within cells. In fact, all groups attenuated the aforementioned changes to varying degrees, but notably, the DOX + HC group displayed significantly superior effect in inhibiting Fe^2+^ accumulation and lipid peroxidation markers compared to the DOX+DA group, suggesting that the anti-ferroptosis effect of M@HCDA was primarily attributable to the HC component, particularly Cel. In summary, these findings confirmed that M@HCDA had excellent anti-DIC activity *in vitro*, effectively alleviating Dox-induced cardiomyocyte injury by inhibiting oxidative stress, reducing lipid peroxidation, and antagonizing ferroptosis.

### 3.3 Injured myocardial targeting verification of M@HCDA on DIC *in vitro* and *in vivo*

Macrophage membrane BNPs typically target inflamed and injured tissues because they retain certain bioactive components on the surface of the source cell membrane. This characteristic can enhance the accumulation of nanomedicines at the lesion site, thereby improving drug delivery efficiency and reducing off-target effects [45], [46]. Given that DIC is accompanied by significant inflammatory responses and the formation of damaged myocardial tissue, this study further evaluates the *in vivo* and *in vitro* targeted delivery capabilities of M@HCDA.

To evaluate cellular uptake *in vitro*, the green fluorescent probe C6 was used to replace the non-fluorescent Cel and siADAM17, yielding targeted NPs M@HD@C6 and non-targeted NPs HD@C6. Fig. 3A and B displayed that M@HD@C6 treatment led to a time-dependent increase in C6 fluorescence intensity and consistently higher than the HD@C6 group at all time points, proving efficient internalization of those NPs by injured cardiomyocytes and enhanced uptake conferred by the macrophage membrane coating. This result also implied that the macrophage membrane endows the NPs with a biological interfacial characteristic for enhancing their targeting ability. The findings were further corroborated by replicate experiments using AC16 cells (Supporting Information Fig. S7A, Fig. 3C) and neonatal rat cardiac fibroblasts (NRCFs, Supporting Information Fig. S7B), which verified that the enhanced uptake effect conferred by macrophage membrane coating applies to injured cardiomyocytes from different sources and to other non-cardiomyocytes in myocardial tissue. Taken together, the biomimetic outer membrane of M@HCDA offered advantages in promoting uptake within the DIC injured cardiomyocytes, providing a foundation for its subsequent role in drug-gene synergistic therapy.

**Fig. 3:**
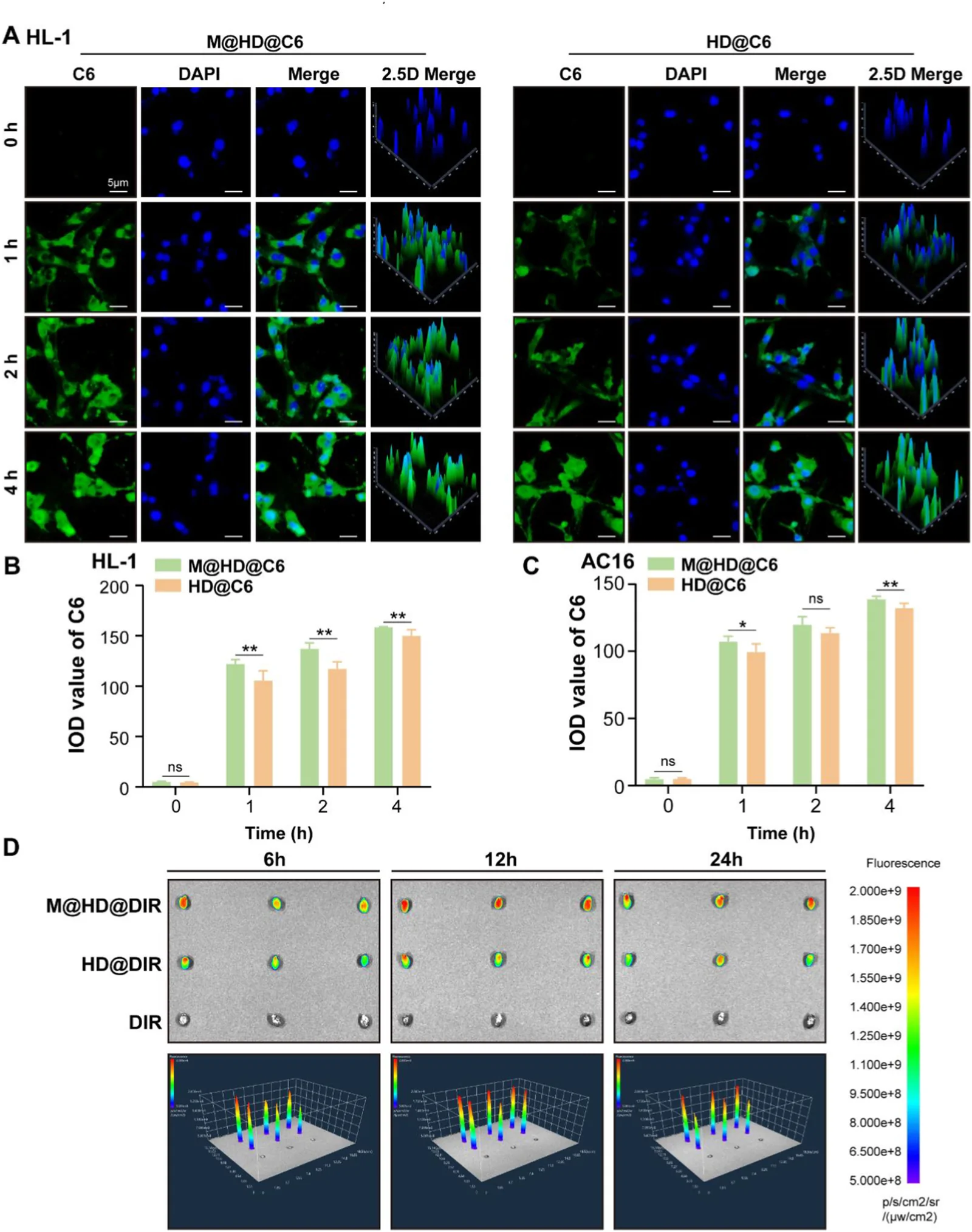
Targeting ability of M@HCDA. Fluorescence images (A) and quantification (B) of uptake of M@HD@C6 and HD@C6 by HL-1 cells at different time points (n = 6). (C) Quantitative analysis of the uptake of M@HD@C6 and HD@C6 by AC16 cells at different time points (n = 6). (D) Fluorescence overlay images of isolated hearts from different groups at 6 h, 12 h, 24 h after injection of respective preparations (n = 3). Scale bar, 5 μm. The data are presented as the mean ± SD; Statistical differences were determined by *t*-test and one-way ANOVA; *p < 0.05, **p < 0.01.

To further evaluate the *in vivo* distribution characteristics of M@HCDA, the near-infrared fluorescent dye DIR was used as a tracer. M@HD@DIR and HD@DIR were prepared, with a free DIR group set as a control. The distribution of NPs in mice was observed by small animal *in vivo* imaging system. *Ex vivo* cardiac imaging at 6 h, 12 h and 24 h after injection revealed that the fluorescence intensity of the M@HD@DIR group remained significantly higher than in the HD@DIR and DIR groups (Fig. 3D, Supporting Information Fig. S8A). It could be concluded that the nano-delivery platform is capable of delivering drugs to damaged myocardial tissue, and that the M@HD@DIR macrophage biomimetic group is significantly superior to the HD@DIR and DIR groups. This result is consistent with *in vitro* uptake experiments, indicating that biomimetic membranes can similarly improve the recognition and accumulation efficiency of NPs in the inflammatory/injured myocardial. Meanwhile, from each group fluorescence distribution results of other isolated organs showed in Supporting Information Fig. S8B. It could be seen from the organ distribution that the fluorescence intensity of the livers, spleens and lungs at each time point was significantly higher than that of the hearts and kidneys. However, the active targeting ability of macrophage membranes proposed in this study mainly refers to that the targeted M@HD@DIR group has a higher nano-drug delivery efficiency to the injured myocardial tissue within the same period of time compared with the non-targeted HD@DIR group and the free DIR group. It was evident from the aforementioned results that macrophage membrane encapsulation enhanced the interaction between NPs and injured myocardial cells but also increased their retention and accumulation levels in the inflammatory myocardial tissue *in vivo*. Compared with the biomimetic membrane-free drugs and free drugs, the lesion-targeting ability of biomimetic membrane improved the delivery efficiency of Cel and siADAM17 to the heart, thus supporting subsequent inhibition of inflammatory responses, alleviation of ferroptosis, and improvement of myocardial injury. In brief, M@HCDA could be efficiently taken up by injured HL-1 and AC16 cells *in vitro* and remarkable accumulate at cardiac lesion tissue *in vivo*, indicating that macrophage membrane coating significantly enhances the NPs delivery advantages to the DIC inflammatory/injured myocardial, thus forming an important foundation for its efficient and synergistic therapy efficacy.

### 3.4 Acute toxicity analysis of M@HCDA

Before further pharmacodynamic studies *in vivo*, it is critical to assess the intravenous administration suitability and safety of M@HCDA. The results displayed that when the concentration of M@HCDA was below 200 μg/mL, the hemolysis rate remained below 5% (Supporting Information Fig. S9), suggesting that this NP exhibits acceptable blood compatibility and meets the basic requirements for subsequent intravenous administration studies. On this basis, the acute *in vivo* safety of M@HCDA was further evaluated through tail vein injection. As shown in Supporting Information Fig. S10A, different doses of M@HCDA (100, 200, and 400 μg/kg) were administered to C57BL/6 mice via tail vein injection, followed by detection and analysis after 24 h. Given that this study primarily focuses on the therapeutic effects of M@HCDA on DIC, it is essential first to exclude potential adverse effects on cardiac function. A small-animal electrocardiogram and ultrasound imaging system were used to assess cardiac function across the various groups. As depicted in Supporting Information Fig. S10B-D, the electrocardiogram rhythms of mice in each group were normal, with stable ST segments and non-significant arrhythmias or ischemic changes. The ultrasound examination also revealed regular cardiac systolic and diastolic activities, with normal mitral valve blood flow velocity. These results suggested that within the dosage range studied, M@HCDA did not induce cardiac dysfunction. Next, H&E staining analysis was performed on the mice major organs of each group, including the heart, liver, spleen, lung, and kidney. The results showed that all the tissues remained structurally intact, with no significant inflammatory cell infiltration, necrosis, or other pathological damage (Supporting Information Fig. S10E), indicating that M@HCDA did not cause acute toxicity in the major organs.

To further assess the systemic biosafety of M@HCDA, the serum samples from all mice were collected to measure the liver and kidney function-related indicators, including aspartate aminotransferase (AST), alanine aminotransferase (ALT), blood urea nitrogen (BUN), and creatinine (CRE). The results revealed that, when the dose of M@HCDA was within 100 - 400 μg/kg, none of the above indicators showed a significant increase across any dosage group (Supporting Information Fig. S10F). Based on above analyses, it can be concluded that M@HCDA is safe for intravenous administration, with no evident acute toxic reactions at doses of 400 μg/kg or lower. Collectively considered, subsequent experiments *in vivo* will choose two dosages, 200 μg/kg and 400 μg/kg.

### 3.5 M@HCDA treats DIC via improving cardiac function, reducing myocardial fibrosis, and alleviating myocardial cell apoptosis

To further evaluate the synergistic treatment activity of M@HCDA *in vivo* via Cel-mediated ferroptosis and siADAM17-mediated anti-inflammatory effects, this study established the C57BL/6 mouse DIC model. The specific experimental procedure is illustrated in Fig. 4A: DOX was administered by intraperitoneal injection at 5 mg/kg once a week; 24 h after each DOX injection, different drug treatments were administered via the tail vein, including M@DA, M@HC, HCDA, and M@HCDA. After four weeks, a series of functional and pathological assessments was conducted. The results of cardiac function showed that compared to the Vehicle group, the DOX group exhibited significant ST-segment elevation on the electrocardiogram; ultrasound results indicated impaired diastolic function, with a marked reduction in left ventricular ejection fraction (EF%) and left ventricular fractional shortening (FS%), alongside a decrease in mitral valve flow velocity (Fig. 4B–D), suggesting that DOX successfully induced cardiac dysfunction. In addition, compared to the DOX group, all treatment groups showed varying degrees of improvement in electrocardiographic and ultrasound abnormalities, with the low-dose group (M@HCDA-L) and high-dose group (M@HCDA-H) exhibiting the most significant improvement, surpassing other treatment groups. Later, myocardial injury serum markers were measured in each group of mice, including creatine kinase isoenzyme (CK-MB), cardiac troponin I (cTnI), and lactate dehydrogenase (LDH). The results revealed that the DOX group showed significant increases in the aforementioned indicators compared to the Vehicle group, highlighting severe myocardial injury. Meanwhile, all drug treatment groups reduced these injury markers to varying degrees, with the M@HCDA group demonstrating the most pronounced improvement (Fig. 4E–G).

**Fig. 4:**
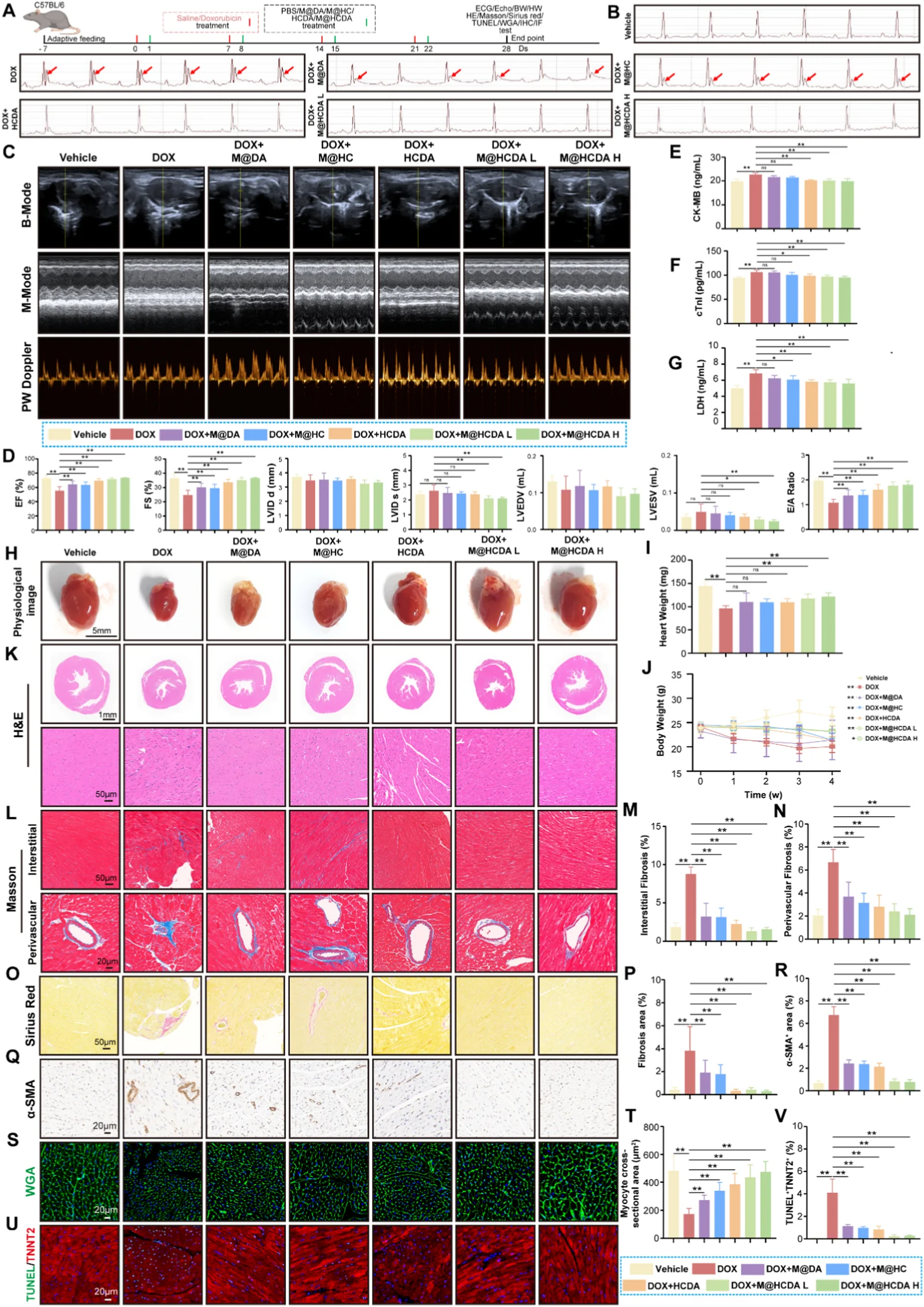
Therapeutic effects of M@HCDA on DIC *in vivo*. (A) Flowchart of *in vivo* pharmacodynamic experiment. Electrocardiograms (B) and B-mode, M-mode, PW Doppler echocardiographic images (C) of each group. (D) The numerical values of EF%, FS%, LVID d, LVID s, LVEDV, LVESV and E/A Ratio in each group. The levels of serum CK-MB (E), cTnI (F), and LDH (G) in each group. (H) Physiological images of mouse hearts from each group. Scale bar, 5 mm. Changes in heart weight (I) and body weight (J) of mice in each group over the four-week period. (K) Overall and local H&E staining images of mouse myocardial tissue in each group. Scale bar, 1 mm and 50 μm. Masson-stained images (L) and quantitative analysis (M, N) of the interstitial and perivascular areas in heart sections from each group of mice. Scale bar, 50 μm and 20 μm. Images (O) and quantification (P) of Sirius Red staining of mouse heart tissues from each group. Scale bar, 50 μm. Immunohistochemical staining images (Q) and quantification (R) of α-SMA in cardiac sections of each group. Scale bar, 20 μm. Images (S) and quantification (T) of WGA staining of cardiac myocytes in each group of mice. Scale bar, 20 μm. Immunofluorescence staining images (U) and quantitative analysis (V) of TUNEL and TNNT2 in myocardial tissue. Scale bar, 20 μm. (n = 6). The data are presented as the mean ± SD; Statistical differences were determined by one-way ANOVA; *p < 0.05, **p < 0.01. (For interpretation of the references to colour in this figure legend, the reader is referred to the Web version of this article.)

At last, all mouse hearts were collected for *ex vivo* imaging and weighing measurement. As shown in Fig. 4H and I, compared to the Vehicle group, both heart volume and weight in the DOX group exhibited a significant reduction. However, the cardiac morphology in both M@HCDA groups was closer to that of the Vehicle group, with heart weight showing a notable increase compared to the DOX group. The body weight changes of mice were continuously monitored throughout the experiment (Fig. 4J), revealing that the weight loss degree was generally consistent with the severity of disease damage. The heart weight/body weight ratio was further calculated (Supporting Information Fig. S11) to assist in evaluating the cardiac atrophy degree. To clarify the histological improvement effect of M@HCDA on DIC myocardial injury, different pathological staining experiments were performed on heart tissue sections from each group of mice. The H&E staining results indicated a significant reduction of heart size in the DOX group (Fig. 4K), consistent with the previously observed *ex vivo* findings (Fig. 4H). High-magnification images further revealed marked inflammatory cell infiltration in the myocardial tissue of the DOX group. Additionally, all groups alleviated these pathological changes to varying degrees, with the M@HCDA group showing the most significant improvement, suggesting its potent anti-inflammatory effect.

Considering that persistent inflammatory responses can further promote myocardial fibrosis, this study employed Masson staining (Fig. 4L–N), Sirius Red staining (Fig. 4O–P), and α-SMA immunohistochemical staining (Fig. 4Q and R) to assess the myocardial fibrosis degree in each group of mice. The results indicated that, compared with the Vehicle group, the DOX group exhibited significant collagen deposition and fibrotic lesions around the myocardial interstitial and perivascular areas. All groups suppressed myocardial fibrosis to some extent compared with the DOX group, with the M@HCDA group showing the best anti-fibrotic effect, followed by the HCDA group, and then the M@DA and M@HC groups. These results suggested that M@HCDA had superior efficacy in improving myocardial fibrosis associated with DIC. The results of WGA staining showed that the cross-sectional area of myocardial cells in the DOX group was significantly reduced, implying substantial cardiomyocyte atrophy. All other groups reversed this change to varying degrees, with the M@HCDA group showing myocardial cell sizes similar to those in the Vehicle group (Fig. 4S and T), further demonstrating its strong protective effect against DOX-induced myocardial structural injury. Finally, to assess the impact of M@HCDA on cardiomyocyte apoptosis, co-staining analysis was performed for the cardiomyocyte marker TNNT2 and TUNEL (Fig. 4U and V). The results denoted a large number of TUNEL-positive cardiomyocyte in the DOX group, indicating a significant increase of cardiomyocyte apoptosis. The M@DA, M@HC, and HCDA groups reduced TUNEL-positive cardiomyocyte to some extent, but both the low- and high-dose M@HCDA groups exhibited very few TUNEL-positive cells, demonstrating the most significant anti-apoptotic effects. Overall, M@HCDA emphasized significant therapeutic effects *in vivo*, effectively improving DOX-induced cardiac dysfunction, alleviating myocardial injury, inhibiting inflammatory responses, and inhibiting myocardial fibrosis and cell apoptosis. And its integrated therapeutic efficacy surpasses that of both the single functional drug and the uncoated composite formulation, suggesting that its cardioprotective effect is mediated by synergistic multiple mechanistic actions.

### 3.6 The therapeutic effects of Mac, Cel, and HD on DIC are limited

To further clarify whether the main pharmacological effects of M@HCDA arose from the Cel and siADAM17, a DIC model *in vivo* was established to conduct verification of relevant components (Fig. 5A). The results of cardiac function showed that, the DOX group exhibited significant cardiac dysfunction. Among the treatment groups, only the HD group showed mild relief, whereas the Mac and Cel groups did not demonstrate improvement (Fig. 5B–D). During the experiment, the survival and body weight of the mice were monitored weekly. The results indicated that the body weights of all mice decreased to varying degrees except for the Vehicle group (Fig. 5E). The survival rate of mice in the Cel group was the lowest at only 20% (Fig. 5F), suggesting that under monotherapy conditions, it did not exhibit ideal cardioprotective effects. The heart weight (Fig. 5G), heart weight/body weight ratio (Supporting Information Fig. S12), *ex vivo* cardiac morphology (Fig. 5H), and H&E staining (Fig. 5I). results indicated that the heart volume and weight of the DOX group mice were significantly reduced, and the three treatment groups did not show improvements in the above indicators. These data pointed to that the administration of these components alone was insufficient to reversed DIC effectively.

**Fig. 5:**
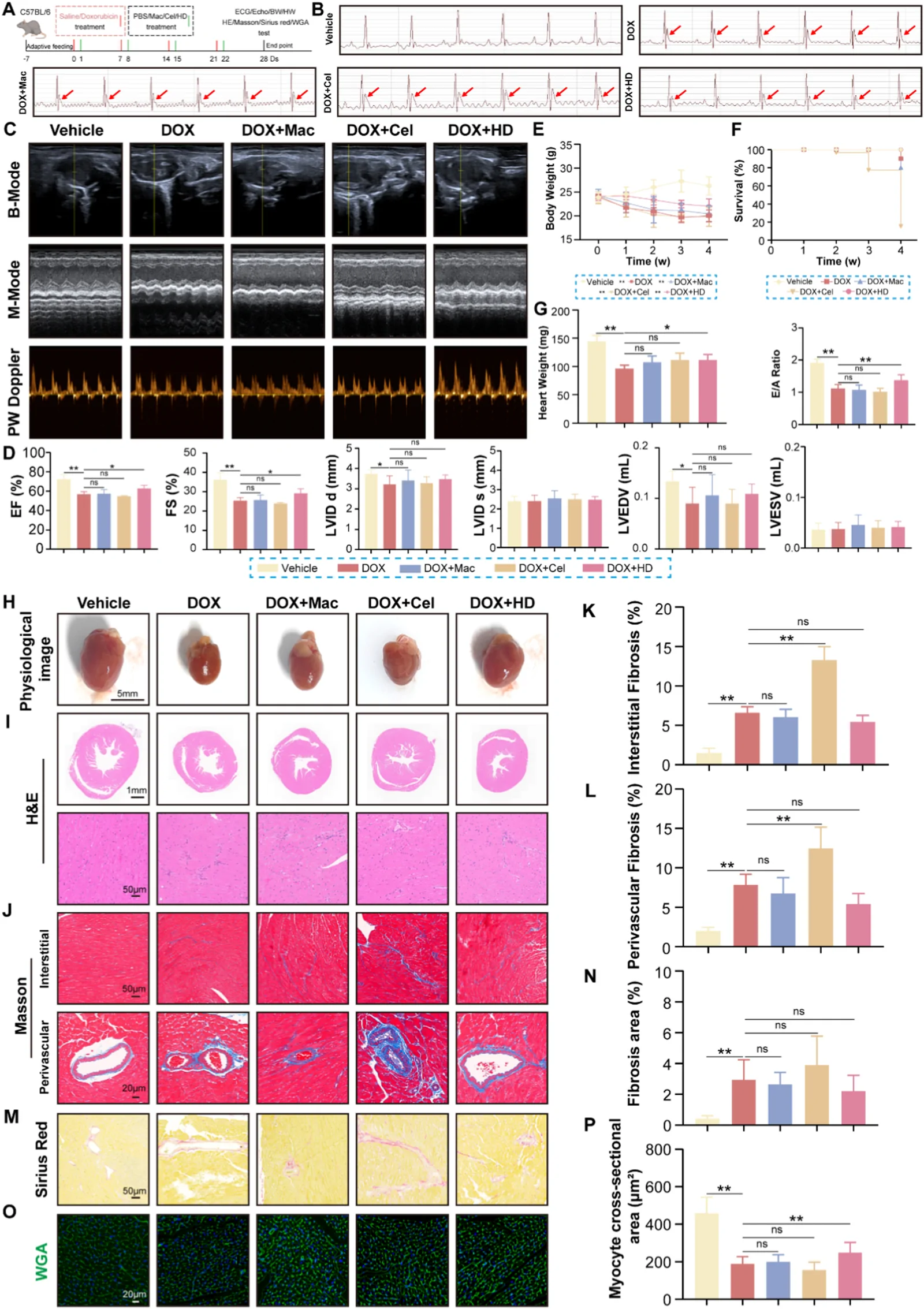
Therapeutic effects of Mac, Cel and HD on DIC. (A) Flowchart of Mac, Cel and HD *in vivo* pharmacodynamic experiment. Electrocardiograms (B) and B-mode, M-mode, PW Doppler echocardiographic images (C) of each group. (D) The numerical values of EF%, FS%, LVID d, LVID s, LVEDV, LVESV and E/A Ratio in each group. Changes in body weight (E) and survival rate (F) of mice in each experimental group over the four-week period, as well as the heart weight (G) of mice in each group at the end. (H) Physiological images of mouse hearts from each group. Scale bar, 5 mm. (I) Overall and local H&E staining images of mouse myocardial tissue in each group. Scale bar, 1 mm and 50 μm. Masson-stained images (J) and quantitative analysis (K, L) of the tissue interstitial and perivascular areas in heart sections from each group of mice. Scale bar, 50 μm and 20 μm. Images (M) and quantification (N) of Sirius Red staining of mouse heart tissues from each group. Scale bar, 50 μm. Images (O) and quantification (P) of WGA staining of cardiac myocytes in each group of mice. Scale bar, 20 μm. (n = 6). The data are presented as the mean ± SD; Statistical differences were determined by one-way ANOVA; *p < 0.05, **p < 0.01. (For interpretation of the references to colour in this figure legend, the reader is referred to the Web version of this article.)

Further, Masson staining (Fig. 5J–L) and Sirius Red staining (Fig. 5M and N) were used to evaluate the effects of each group on myocardial fibrosis. The results showed that the Mac, Cel and HD groups did not exhibit anti-fibrotic effects compared with the DOX group. Analysis of cardiomyocyte size using WGA staining revealed that only the HD group showed a slight increase in cardiomyocyte cross-sectional area, and the Mac and Cel groups had no significant improvements (Fig. 5O and P). These results indicated that membrane components or carrier materials played only an auxiliary role, and could not achieve the significant therapeutic effects demonstrated by M@HCDA. Notably, it was evident from the above results that the myocardial injury degree in the Cel group was higher than that in the DOX group. This outcome may result from poor systemic absorption and utilization of the insoluble drug following intravenous administration, thereby exacerbating cardiac load. In previous studies on the effects of Cel against cardiomyopathy, intraperitoneal injection of Cel with 0.1% DMSO was used, and Cel showed significant preventive and therapeutic effects [32], [47]. However, in this study, the group receiving tail vein injection of Cel with PBS was selected as the control group to clarify the specific therapeutic effects distinct from the nanodrug group, which may be the main reason why the myocardial injury in the Cel group were more obvious than that in previous studies. To conclude, the anti-DIC efficacy of M@HCDA *in vivo* was primarily attributable to its encapsulated active components rather than to the macrophage membrane or the lipid carrier. The fact none of the individual components afforded cardioprotection comparable to M@HCDA, confirming that the substantial therapeutic efficacy of this nanocarrier arises from synergistic effects among its constituents rather than from any single component alone.

### 3.7 Chronic toxicity analysis of M@HCDA

The chronic toxicity analysis was undertaken to evaluate the biocompatibility and safety of the M@HCDA BNPs *in vivo*, given that DOX could induced cardiotoxicity and potentially damage other vital organs, such as the liver and kidney. Mice were received tail vein injections of M@HCDA (400 μg/kg) once weekly for 4 weeks, followed by functional and pathological assessments (Supporting Information Fig. S13A), including electrocardiograms, ultrasound imaging, H&E staining of major organs, and detection of liver and kidney function (Supporting Information Fig. S13B-F). These results showed no significant differences in physiological functions or histological indicators between the vehicle and M@HCDA groups, indicating that M@HCDA had great biocompatibility *in vivo*.

### 3.8 mRNA-sequencing analysis in the treatment of DIC with M@HCDA

To explored the intrinsic mechanisms of M@HCDA against DIC and to uncovered transcriptional changes in mice following treatment, this study conducted RNA sequencing of myocardial tissues from three groups: Vehicle, DOX, and DOX + M@HCDA. The principal component analysis (PCA) results indicated significant differences in the distribution among the three groups (Fig. 6A). Pairwise comparisons among the three groups yielded three sets of differential gene enrichment analyses: DOX versus (vs) Vehicle, DOX + M@HCDA vs DOX, and DOX + M@HCDA vs Vehicle. Given that this experiment primarily investigates the therapeutic effects of the M@HCDA formulation on DIC, the focus will be on the first two sets of differential analyses. Based on this, a series of analyses of DEGs was conducted with log_2_|FC| ≥ 0.5 and P < 0.05. As shown in Fig. 6B and C and Supporting Information Fig. S14A-B, the volcano plots and heatmaps of the front two pairs of DEGs analyses. The Venn diagram (Fig. 6D) was generated from the two pairs of DEGs analyses, indicating that the genes which M@HCDA exerts anti-DIC effects may be among the 206 co-expressed genes. Gene Ontology (GO) and Kyoto Encyclopedia of Genes and Genomes (KEGG) enrichment analyses were performed on the up- and down-regulated DEGs from both pairs. As showed in the GO enrichment analysis, the injury caused by DOX to myocardium was primarily manifested as an upregulation of “collagen fibril organization” (Fig. 6E). Meanwhile, trends of upregulation were also observed for “superoxide metabolic,” “iron ion homeostasis,” “inflammatory cell apoptosis,” and “mitochondrial depolarization”. Correspondingly, the negative regulation of these functions showed downregulation trend. The KEGG enrichment analysis yielded the same conclusions. Notably, it also showed a significant increase in enrichment of the “Ferroptosis” and “MAPK signaling pathway” pathways following DOX intervention (Fig. 6F). On the other hand, when comparing the DOX + M@HCDA group with the DOX group, functions related to antioxidant, anti-apoptotic, and mitochondrial repair showed upregulation (Fig. 6G). According to Fig. 6H, the “MAPK signaling pathway” displayed a downregulation trend, suggesting that M@HCDA may exerts anti-DIC effects through it. To better illustrate the mechanism of M@HCDA in DIC, gene set enrichment analysis (GSEA) was performed. The results of the GSEA-GO analysis for DOX vs Vehicle indicated that related enrichment analyses for pro-inflammatory, pro-oxidative stress, pro-apoptotic, pro-fibrotic, pro-ferroptotic, and pro-mitochondrial dysfunction pathways were all highly expressed (Fig. 6I–L), further demonstrating that DOX-induced cardiotoxicity was multifaceted and complex. Moreover, the GSEA-GO analysis results for DOX + M@HCDA vs DOX revealed that the related enrichment analyses for above various pathways were all lowly expressed (Fig. 6M–P). In the subsequent GSEA-KEGG enrichment analysis, pathways related to “Ferroptosis”, "MAPK pathway", and "Apoptosis" were also enriched under disease conditions. Pathways such as "TCA cycle", "Fatty acid metabolism", and "AMPK pathway" were upregulated after M@HCDA treatment (Fig. 6Q and R). Regarding the ferroptosis pathway, the expression of GPX4 and SLC7A11 were statistically analyzed each group (Supporting Information Fig. S15). Additionally, GSEA cnetplot displayed the relevant genes involved in each pathway (Supporting Information Fig. S16, S17). In general, it was evidenced from the above analyses that the expression and functional enrichment of genes related to the "MAPK signaling pathway" and "Ferroptosis" were significantly upregulated following DOX intervention and markedly downregulated after M@HCDA treatment, implying that these two signaling pathways may be the primary mechanisms which M@HCDA against DIC. As shown in Fig. 6S, it could be observed that treatment with M@HCDA also alleviated the expression of some myocardial injury marker genes.

**Fig. 6:**
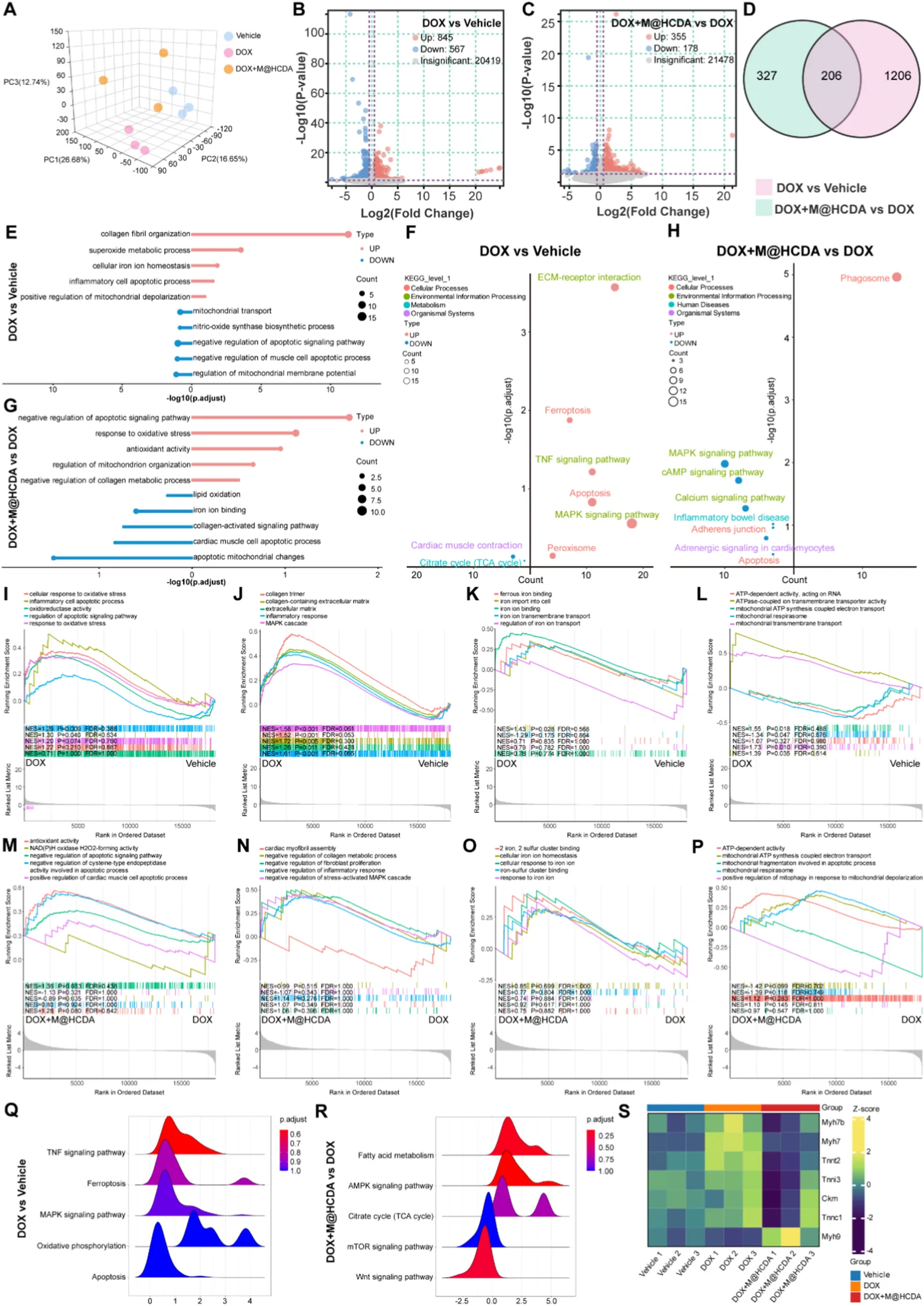
RNA sequencing analysis of M@HCDA anti-DIC. (A) 3D PCA plot of three groups of samples: Vehicle, DOX, and DOX + M@HCDA groups. Volcano plots of DEGs for the comparison between DOX vs Vehicle (B) and DOX + M@HCDA vs DOX (C), with thresholds set at Log_2_|FC| ≥ 0.5 and P value < 0.05. (D) Venn diagram of the combined two sets of DEGs. (E, G) Lollipop plots of GO upregulated and downregulated enrichment analysis of DEGs in two sets. (F, H) Scatter plots of KEGG pathway upregulated and downregulated enrichment analysis of DEGs in two sets. Based on the DEGs of DOX vs Vehicle and DOX + M@HCDA vs DOX, perform GSEA-GO enrichment analysis for functions and pathways related to oxidative stress and apoptosis (I, M), inflammation and fibrosis (J, N), ferroptosis (K, O), and mitochondrial homeostasis (L, P). (Q-R) Ridge plots of GSEA-KEGG enrichment analysis based on DEGs from two sets. (S) Heatmap of gene expression levels for each sample of markers of myocardial injury.

### 3.9 M@HCDA treats DIC via inhibiting ferroptosis and the MAPK signaling pathway to improve mitochondrial dysfunction

To further elucidated the relationship between the Cel and ferroptosis pathway, GPX4 (PDB ID: 2OBI) and SLC7A11 (PDB ID: 7P9V) were selected as ferroptosis representative indicators to predict interactions with Cel via molecular docking and molecular dynamics (MD) simulations [48], [49]. Fig. 7A and B illustrated the predicted interaction between Cel and GPX4 or SLC7A11, revealing that Cel could interacted with multiple sites through various intermolecular forces, with the binding affinity of −7.312 kcal/mol and −8.904 kcal/mol respectively. The RMSD (Fig. 7C and D), RMSF (Fig. 7E and F) and radius of gyration (Rg) (Fig. 7G and H) of GPX4/Cel and SLC7A11/Cel complexes in MD indicated that, Cel binding did not appreciably disrupt the whole protein structural stability, instead, the stability of the complexes was better than that of the protein monomer. By combining RMSD and Rg data, the 3D free energy landscape (FEL) for each complex could be derived. The results consistently revealed a single, deep energy well, indicating that the system continuously in a stable low-energy conformational region during the simulation (Fig. 7I and J). This finding indicated that the GPX4/Cel and SLC7A11/Cel complexes exhibited high conformational stability. Based on the above, it was posited that Cel could stably binding to GPX4 and SLC7A11, enhancing complex stability by maintaining the protein structure compactness, suggesting Cel could exert anti-ferroptosis effects by preventing the degradation of GPX4 and SLC7A11.

**Fig. 7:**
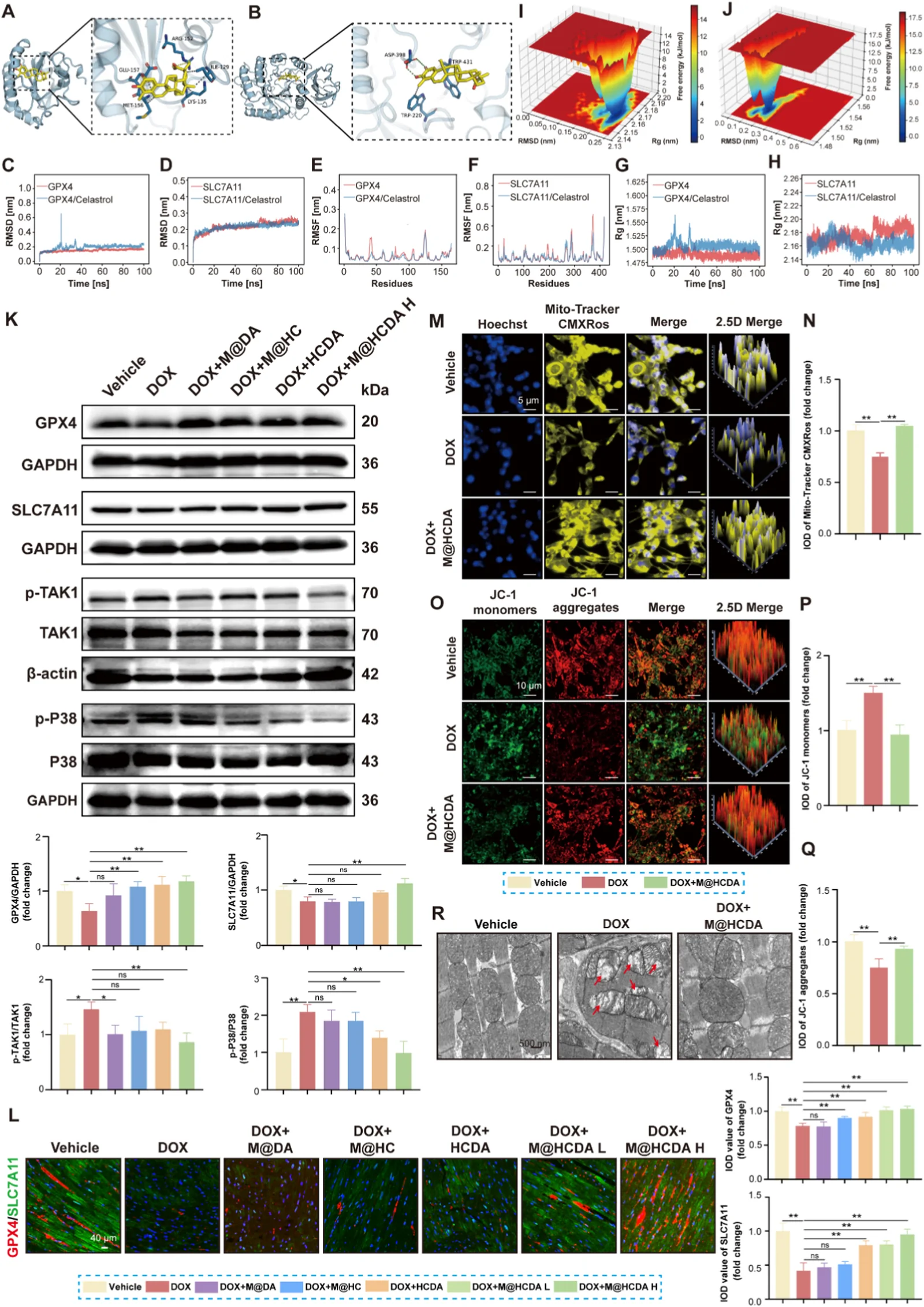
The mechanism of M@HCDA in treating DIC. (A) Binding mode diagram of the GPX4/Cel complex. (B) Binding mode diagram of the SLC7A11/Cel complex. Protein GPX4 and GPX4/Cel complex RMSD (C), RMSF (E) and Rg (G) in MD. Protein SLC7A11 and SLC7A11/Cel complex RMSD (D), RMSF (F) and Rg (H) in MD. 3D FEL analysis of GPX4/Cel complex (I) and SLC7A11/Cel (J). (K) Immunoblotting analysis of GPX4, SLC7A11, p-TAK1, TAK1, p-P38, P38 protein levels in the hearts (up) and quantification (down, n = 3). (L) Immunofluorescence staining images and quantification of GPX4 and SLC7A11 in myocardial tissue (n = 6). Scale bar, 40 μm. Mito-Tracker CMXRos staining (M) and quantitative analysis (N), JC-1 staining (O) and quantitative analysis (P, Q) in HL-1 cells after treatment with M@HCDA (n = 6). Scale bar, 5 μm and 10 μm. (R) Bio-TEM images of myocardial tissue in the three groups after the *in vivo* experiment. Scale bar, 500 nm. The data are presented as the mean ± SD; Statistical differences were determined by one-way ANOVA; *p < 0.05, **p < 0.01.

Afterwards, this study employed Western blot analysis to elucidated how M@HCDA in regulates ferroptosis and the TAK1-MAPK signaling pathway and to detect the relative protein expression levels. Before this, we detected the changes in ADAM17 mRNA and protein levels in mouse cardiac tissue. The efficiency of ADAM17 silencing is crucial for downstream pathway expression. The results showed that compared with the Vehicle group, the ADAM17 level in the DOX group was significantly increased, and there was a decrease to varying degrees in each treatment group, with the most significant decrease in the DOX + M@HCDA H group (Supporting Information Fig. S18A). The same conclusion was also obtained from the mRNA level (Supporting Information Fig. S18B). As shown in Fig. 7K, compared with the Vehicle group, the DOX group exhibited decreased levels of GPX4 and SLC7A11, along with increased levels of p-TAK1 and p-P38. Although each group demonstrated alleviation to varying degrees, the protein expression level in the DOX + M@HCDA group was nearly indistinguishable from those in the Vehicle group. To further clarify the mechanism by which M@HCDA inhibits ferroptosis, this study treated damaged HL-1 cells with RSL3 (a GPX4 inhibitor) and then verified GPX4 levels (Supporting Information Fig. S19). It was found that RSL3 could significantly inhibit the up-regulation of GPX4 induced by M@HCDA. It can be concluded that this further confirms that M@HCDA exerts its anti-ferroptosis effect by stabilizing GPX4/SLC7A11. As illustrated in Fig. 7L, the immunofluorescence expression levels of GPX4 and SLC7A11 were consistent with the Western blot results. Additionally, previous RNA sequencing analyses indicated that M@HCDA had a certain alleviating effect on DOX-induced mitochondrial dysfunction. The typical mitochondrial damage characteristics associated with ferroptosis include increased membrane density and even rupture, as well as the reduction or disappearance of mitochondrial cristae [50]. As shown in Fig. 7M and N, the HL-1 cell staining results with Mito-Tracker CMXRos, revealing that the fluorescence intensity in the DOX group was significantly reduced, and the DOX + M@HCDA group markedly reversed this effect. JC-1 staining results showed that the expression level of JC-1 monomers increased, while the corresponding expression level of JC-1 aggregates decreased in the DOX group. In contrast, the M@HCDA group showed an opposite result to that of the DOX group, demonstrating that its mitochondrial membrane potential was restored to normal (Fig. 7O–Q). Bio-TEM analysis showed that mitochondria vacuolation and cristae were absented, with an irregular arrangement of myocardial fibres in the DOX group. In the DOX + M@HCDA group, the vacuolation of mitochondria was significantly alleviated, and the distribution of myocardial fibers was consistent with that of the Vehicle group (Fig. 7R). Another core characteristic of ferroptosis is lipid peroxidation. As shown in Supporting Information Fig. S20, the MDA level in the mice of the DOX group was significantly higher than that in the Vehicle group, and other groups showed varying degrees of alleviation. In brief, M@HCDA could treated DIC and alleviated mitochondrial dysfunction by regulating the MAPK and ferroptosis pathways.

## 4 Conclusion

This study successfully designed and constructed macrophage membrane BNPs M@HCDA for the co-delivery of Cel and siADAM17 in DIC therapy. *In vitro* and *in vivo* experiments demonstrated that M@HCDA significantly alleviated DOX-induced myocardial injury, enhanced cardiomyocyte viability, inhibited ROS generation, Fe^2+^ accumulation, and mitigated lipid peroxidation, cardiac dysfunction, myocardial fibrosis, cardiomyocyte injury, and apoptosis. Mechanistic studies further confirmed that the Cel encapsulated in M@HCDA exerted an anti-ferroptosis effect by targeting and regulating GPX4 and SLC7A11, whereas siADAM17 inhibited TAK1-MAPK signaling by silencing ADAM17, thereby exerting anti-inflammatory and anti-fibrotic effects, and synergistically improving mitochondrial dysfunction. Meanwhile, M@HCDA exhibited excellent stability and biosafety, and achieved targeted delivery to the injured myocardium, effectively delivering drugs and genes to the injury site. In conclusion, the M@HCDA achieved effective DIC therapy through multi-target synergistic intervention, providing new experimental evidence for the prevention and treatment of DIC and offering a theoretical basis for the application of biomimetic nano systems in cardiovascular diseases.

## CRediT authorship contribution statement

**Qiyu Zhang:** Writing – original draft, Visualization, Methodology, Investigation. **Tianyu Guo:** Visualization, Methodology, Investigation. **Xuna Xue:** Validation. **Zhibo Zhang:** Validation. **Xinyan Liu:** Validation. **Qing Cai:** Validation. **Ning Wang:** Writing – review & editing, Writing – original draft, Supervision. **Bo Zhang:** Writing – review & editing, Supervision. **Jinyong Peng:** Writing – review & editing, Supervision.

## Declaration of competing interest

The authors declare that they have no known competing financial interests or personal relationships that could have appeared to influence the work reported in this paper.

## Data Availability

Data will be made available on request.
