# Supplementary material for: Targeted delivery of celastrol and siRNA via macrophage biomimetic nanoparticles for doxorubicin-induced cardiomyopathy therapy
Source: Mater Today Bio. 2026 Oct 23;41:103779. doi: 10.1016/j.mtbio.2026.103779 (PMC13722028; doi:10.1016/j.mtbio.2026.103779)
Supplement: Supplementary file 1 [file mmc1.docx]

**Supporting information**

**Supporting methods**

- **Synthesis of HA-TK-PEG-Chol**

From our previous work, COOH-TK-COOH and Chol-NH_2_ were obtained [1].

HA (1.2 g) was dissolved in 150 mL dimethyl sulfoxide (DMSO). Then, EDCI (0.69 g, 3.6 mmol), DMAP (0.44 g, 3.6 mmol) and COOH-TK-COOH (0.91 g, 3.6 mmol) were added. After stirring at 40℃ for 24 hours, the product was purified by dialyzing in a membrane (MWCO 8-14k Da) against deionized for 72 h, filtered, centrifuged and lyophilized to obtain the white product HA-TK-COOH (1.24 g). The product structure was characterized by ^1^H NMR.

HA-TK-COOH (0.42 g) was dissolved in 100 mL dimethyl sulfoxide (DMSO). Next, EDCI (0.46 g, 2.4 mmol), NHS (0.28 g, 2.4 mmol) and NH_2_-PEG-NH_2_ (2.4 g, 2.4 mmol) were added. After stirring at 40℃ for 24 hours, the product was purified by dialyzing in a membrane (MWCO 8-14k Da) against deionized for 96 h, filtered, centrifuged and lyophilized to obtain the white product HA-TK-PEG-NH_2_ (1.26 g). The product structure was characterized by ^1^H NMR.

Chol-NH_2_ (0.94 g, 2 mmol) was dissolved in 50 mL CHCl_3_. Then, COOH-TK-COOH (0.51 g, 2 mmol), EDCI (0.38 g, 2 mmol) and NHS (0.22 g, 2 mmol) were added. After stirring at RT for 24 hours, the reaction solution was washed with brine and water, respectively. The organic phase was concentrated to obtain a white crude product. The crude product was purified using silica gel chromatography (CHCl_3_/CH_3_OH=12:1) to obtain 0.76 g of white solid Chol-TK-COOH (yield 54.1%). The product structure was characterized by ^1^H NMR and HRMS. ^1^H NMR (500 MHz, DMSO-*d_6_*): δ/ppm = 5.32 (s, 1H); 4.30 (s, 1H); 3.60 (s, 2H); 3.50 (s, 1H); 3.31 (m, 6H); 3.16 (m, 1H); 3.05 (m, 1H); 3.01 (t, 3H); 2.79 (m, 8H); 2.60 (m, 2H); 2.24 (m, 3H); 1.97 (m, 2H); 1.79 (m, 2H); 1.53 (m, 11H); 1.23 (m, 6H); 1.05 (m, 8H); 0.86 (m, 6H); 0.65 (s, 2H). HRMS: M=706.44131; m/z=705.43530 [M-H]^-^.

HA-TK-PEG-NH_2_ (0.58 g) was dissolved in 100 mL dimethyl sulfoxide DMSO). Then, EDCI (0.23 g, 1.2 mmol), NHS (0.14 g, 1.2 mmol) and Chol-TK-COOH (0.85 g, 1.2 mmol) were added. After stirring at 40℃ for 24 hours, the product was purified by dialyzing in a membrane (MWCO 8-14k Da) against deionized for 72 h, filtered, centrifuged and lyophilized to obtain the white product HA-TK-PEG-Chol (0.84 g). The product structure and molecular weight was characterized by ^1^H NMR, DOSY and GPC, respectively.

- **Characterization of HCDA and M@HCDA**

The size distribution, zeta potential and morphology of HCDA and M@HCDA were characterized through dynamic light scattering (DLS, Malvern Zetasizer Nano ZS) and transmission electron microscopy (TEM, JEM-2000EX*, Japan).

- **Cytotoxicity study** **in HL-1 and AC16 cells**

HL-1 and AC16 cells were seeded in 96-well plates at a specific density and cultured overnight at 37°C in 5% CO₂ atmosphere. Subsequently, fresh culture media containing M@HCDA at concentrations of 0 nM, 1 nM, 10 nM, 20 nM, 40 nM, 60 nM, 80 nM, and 100 nM were added respectively and the cells were further cultured for 24 hours. After that, 10 μl of CCK-8 reagent was added to each well and incubated for 15 - 45 minutes. The absorbance was measured at 450 nm using a microplate reader.

The CCK-8 method was used to evaluate the cell viability of HL-1 and AC16 cells after damage caused by DOX. Cell seeding was carried out as described above. Subsequently, fresh culture media containing DOX at concentrations of 0 μM, 0.25 μM, 0.5 μM, 1 μM, 2 μM, and 4 μM were added respectively and the cells were further cultured for 24 hours. Then, CCK-8 reagent was added and the absorbance was measured.

The CCK-8 method was used to evaluate the changes in cell viability after the intervention of multiple drugs such as PBS, macrophages (Mac), HD, Cel, DA, HC, HCDA, and M@HCDA in combination with DOX. Cell seeding was carried out as described above. Subsequently, fresh culture media containing different drugs at the same concentration were added respectively and the cells were further cultured for 24 hours. Then, CCK-8 reagent was added and the absorbance was measured.

To investigate the inhibitory effect of small interfering RNA (siRNA) on ADAM17 in HL-1 cells, siRNA targeting ADAM17 (siADAM17-1, sense strand: 5’- GAGAGCACC-ACUACAAAUUTT -3’, antisense strand: 5’- AAUUUGUAGUGGUGCUCUCTT -3’; siADAM17-2, sense strand: 5’- CCUCUCCUUAGCUAAUAUUTT -3’, antisense strand: 5’- AAUAUUAGCUAAGGAGAGGTT -3’) was used. The siRNA targeting ADAM17 was transfected into HL-1 cells for 24 hours using Lipo2000 Transfection Reagent (Glpbio Technology Inc, CA, USA). The expression level of ADAM17 was assessed by Western blotting.

- **Live/Dead cell staining assay in HL-1 and AC16 cells**

HL-1 and AC16 cells were seeded in 6-well plates at a specific density and cultured overnight at 37°C in 5% CO₂ atmosphere. Subsequently, fresh culture media containing M@HCDA at concentrations of 0 nM, 1 nM, 10 nM, 20 nM, 40 nM, 60 nM, 80 nM, and 100 nM were added respectively, and the cells were further cultured for 24 hours. After that, the cells were washed three times with pre-cooled PBS. Then, the cells were stained using the Calcein/PI Live/Dead Assay Kit and observed under an inverted fluorescence microscope (Zeiss, Oberkochen, Germany).

- **Determination of ROS, iron ion and** **lipid peroxidation levels**

The BioTracker 575 red Fe^2+^ dye was used to detect the Fe^2+^ level *in vitro*, the DCFH-DA probe was used to detect the ROS level *in vitro* and the C_11_-BODIPY probe was used to detect the lipid peroxidation level *in vitro*. HL-1 cells were seeded in 6-well plates at a specific density and cultured overnight at 37°C in 5% CO_2_ atmosphere. The next day, fresh culture media containing drugs such as DA, HC, HCDA, and M@HCDA at a concentration of 20 nM were added to each drug treatment group respectively. An equal volume of PBS was added to the vehicle and DOX groups, and then they were incubated for another 24 hours. The next day, after removing the original culture medium, except for the vehicle group, fresh culture medium containing 1 μM DOX was added to the other experimental groups, and an equal volume of PBS was added to the vehicle group for continuous culture for another 24 hours. Subsequently, the cells were washed three times with pre-cooled PBS, stained with the BioTracker 575 red Fe^2+^ dye, the DCFH-DA probe and the C_11_-BODIPY probe respectively, and observed under an inverted fluorescence microscope.

- **Detection of *in vitro* mitochondrial-related indicators**

The experiment was divided into three groups: Vehicle, DOX, and DOX + M@HCDA. As previously described, cell seeding, drug intervention, and DOX intervention were carried out. After washing the cells three times with pre - cooled PBS, the cells were stained with the enhanced mitochondrial membrane potential assay kit with JC-1 and Mito-Tracker Red CMXRos respectively, and observed under an inverted fluorescence microscope.

- ***In vivo* acute toxicity experiments**

Twenty-four male C57BL/6 mice were randomly divided into four groups: the vehicle group and three groups treated with different doses of M@HCDA (100 μg/kg, 200 μg/kg, and 400 μg/kg). Mice in the M@HCDA group were injected with the corresponding dose of M@HCDA via the tail vein, while mice in the Vehicle group were injected with an equal volume of PBS. After 24 hours, the mice were anesthetized with isoflurane, and cardiac function was evaluated using the small animal ultrasound detection system (Yeeran, Beijing, China) and the small animal electrocardiogram. The left ventricular ejection fraction (EF%), fractional shortening (FS%), left ventricular internal diameter at end - diastole (LVID d), left ventricular internal diameter at end-systole (LVID s), left ventricular end-diastolic volume (LVEDV), left ventricular end-systolic volume (LVESV), and E/A ratio were calculated for all mice. Subsequently, the mice were euthanized, and whole blood and major metabolic organs were collected for biochemical analysis and H&E staining.

- ***In vivo* chronic toxicity experiments**

Twelve male C57BL/6 mice were randomly divided into two groups: the Vehicle group and the M@HCDA group (400 μg/kg). The experiment lasted for a total of four weeks, with injections administered once a week. Mice in the M@HCDA group were injected via the tail vein at a dose of 400 μg/kg, while mice in the Vehicle group were injected with an equal volume of PBS. After four weeks, a series of subsequent analyses of cardiac function, blood biochemistry, and H&E staining were performed as previously described.

- **Cellular uptake** **in HL-1 and AC16 cells**

As previously described, *in vitro* cell uptake experiments were performed using M@HD@C6 and HD@C6 NPs. HL-1 and AC16 cells were inoculated in 6-well plates at a specific density and cultured overnight in a 5% CO_2_ atmosphere at 37℃. After removing the culture medium, fresh DMEM medium containing 1 μM DOX was added and continue cultured for 24 hours. After removing the medium containing DOX, the medium containing HD@C6 and M@HD@C6 were added respectively, and cultured for 0, 1, 2, and 4 hours respectively. Finally, the culture medium was discarded, and the cells were washed three times with cold PBS. The nuclei of the cells were then stained with DAPI for 10 minutes and observed using inverted fluorescence microscopy.

- **Small animal *in vivo* imaging study**

As mentioned previously, *in vivo* imaging experiments were conducted using M@HD@DIR and HD@DIR NPs. Nine male C57BL/6 mice were randomly divided into three groups: the M@HD@DIR group, the HD@DIR group, and the DIR group. The experiment lasted for a total of four weeks, and DOX (5 mg/kg) was intraperitoneally injected once a week. After four weeks, mice in each experimental group were injected with the corresponding nanoparticles via the tail vein at a dose of 1 mg/kg, and *in vivo* imaging was performed at different time points using a small animal *in vivo* imaging system (Biolight, Guangzhou, China). Twenty-four hours later, the mice were euthanized, and the hearts were collected for fluorescence imaging to evaluate the targeting differences among the three groups.

- ***In vivo* pharmacodynamic evaluation of anti-DOX-induced cardiomyopathy**

To investigate the effect of M@HCDA against doxorubicin-induced cardiomyo -pathy, the in vivo experiment was divided into seven experimental groups, namely Vehicle, DOX, DOX+DA, DOX+HC, DOX+HCDA, DOX+M@HCDA L, and DOX+M@HCDA H. The experiment lasted for a total of four weeks. DOX (5 mg/kg) was intraperitoneally injected once a week, and various drugs were injected via the tail vein on the day after DOX injection. The body weight of the mice was monitored weekly. After four weeks, cardiac function was detected by echocardiography and electrocardiogram in the mice as previously described. Subsequently, the mice were anesthetized with tribromo-ethanol, and arterial blood and hearts were collected. After weighing the hearts and taking photos of the specimens, the hearts were fixed in 4% paraformaldehyde in batches and stored at -80°C for future use.

- ***In vivo* pharmacodynamic evaluation of anti-DOX-induced cardiomyopathy**

To investigate the effects of macrophage membranes, free celastrol, and pure nanomaterials on DOX-induced cardiomyopathy, the in vivo experimental part was divided into five experimental groups, namely Vehicle, DOX, DOX+Mac, DOX+Cel, and DOX+HD. As previously described, DOX injection and nanodrug intervention were carried out in the in vivo experiment. After four weeks, the cardiac function of the mice was detected, and then arterial blood and hearts were collected. After weighing the hearts and taking photos of the specimens, they were fixed in 4% paraformaldehyde in batches.

- **Pathological staining**

The above myocardial tissues fixed with 4% paraformaldehyde were subjected to gradient dehydration and paraffin embedding, and then cut into 4-μm tissue slices and adhered to glass slides for later use. According to the experimental requirements, H&E staining, Masson staining, Sirius red staining, α-SMA immunohistochemical staining, WGA staining, TUNEL staining, collagen I/collagen III immunofluorescence double-labeling staining, GPX4/SLC7A11 immunofluorescence double-labeling staining, etc. were performed in sequence.

- **Bio-electron microscopy**

After the in vivo pharmacodynamic experiment, three groups of mice, namely the Vehicle, DOX, and DOX + M@HCDA H groups, were anesthetized with tribromoethanol. Then, the hearts were removed and placed in cold PBS for free pumping until no obvious blood flowed out. The rinsed myocardial tissues were cut into squares with a width of 1-2 mm using the scalpel, immersed in the electron microscope fixative for fixation, and observed under a biological electron microscope.

- **mRNA-sequencing analysis**

Total RNA was extracted from myocardial tissues of the Vehicle group (n = 3), DOX group (n = 3), and DOX + M@HCDA H group (n = 3) using TRIzol (Invitrogen, USA) according to the manufacturer's instructions. RNA purification, reverse transcription, library construction, and sequencing were performed at Metware Biotechnology Co., Ltd. in Wuhan, China.

- **Molecular docking and molecular dynamics simulation**

The crystal structure of the target protein and the 3D structure of the small molecule were obtained from the PDB database and the PubChem database. Before docking, operations such as removing water molecules, salt ions, and small molecules from the target protein were performed using PyMol 2.5.5. AutoDock Vina 1.1.2 was applied for molecular docking. Subsequently, the conformation with the highest score was selected and imported into AMBER 18 for molecular dynamics simulation. The binding free energy between the protein and the ligand in all systems was calculated by the molecular mechanics/Poisson - Boltzmann surface area method.

- **Western blotting**

Proteins were extracted from the above-mentioned myocardial tissues stored at -80°C using RIPA buffer and PMSF protease inhibitor, and were quantified to 50 μg using a BCA assay kit. Subsequently, through SDS-PAGE gel electrophoresis, the membranes were incubated overnight at 4°C with primary antibodies against p-TAK1, TAK1, p-P38, P38, GPX4, and SLC7A11 respectively. Then incubated with appropriate goat anti-rabbit/mouse secondary antibodies the next day. All blots were analyzed using Image J software, with GAPDH or β-actin as internal references.

Extract the proteins from the lysates of macrophage cell membranes, nuclei, cytoplasm and whole cells as described above, and then verify the components through protein gel electrophoresis experiments. Incubate the membranes with primary antibodies such as the cell membrane markers (F4/80 and CD68), the cytoplasmic marker (LDHA + LDHB), and the nuclear marker (Lamin A/C) respectively, and then incubate with appropriate goat anti - rabbit/mouse secondary antibodies.

- **Hemolysis test**

Dilute the blood of male C57BL/6 mice 30-fold with normal saline and add it to a 24-well plate at a volume of 1 mL per well. Add M@HCDA solutions of different concentrations (50, 100, 200, 300, 400 μg/mL) and incubate at 37 °C for 4 hours. After incubation, transfer 0.9 mL of the blood solution to a centrifuge tube and centrifuge at 2000 rpm for 5 min to remove intact red blood cells. Subsequently, transfer 200 μL of the supernatant to a 96-well plate and measure the absorbance at 540 nm using a microplate reader. Red blood cells treated with normal saline served as a negative control. Red blood cells treated with 1% Triton X-100 are used as a positive control.

**References**

1 M. Gao, L. Yin, B. Zhang, Z. Dong, W. Jiang, Z. Bai, et al., Targeting Ischemic Myocardium: Nanoparticles Loaded with Long Noncoding RNA AK156373 siRNA Alleviate Myocardial Infarction, ACS Nano 19 (2025) 18475–18491. https:// doi.org/10.1021/acsnano.5c01641.

**Table. S1** Summary Table of Nanoparticle Composition

| NPs | Components and proportions | Mass ratio |
| --- | --- | --- |
| HC  DD  DA | HA-TK-PEG-Chol/Cel  DOTAP/DOPE  DOTAP/DOPE/siADAM17 | 3:1  1:1  5:5:1 |
| HCDD | HA-TK-PEG-Chol/Cel/DOTAP/DOPE | 3:1:5:5 |
| HCDA | HA-TK-PEG-Chol/Cel/DOTAP/DOPE/siADAM17 | 3:1:5:5:1 |
| M@HCDA | Macrophage membrane/HA-TK-PEG-Chol/Cel/DOTAP/DOPE/siADAM17 | 15:3:1:5:5:1 |
| HD@C6 | HA-TK-PEG-Chol/ DOTAP/DOPE/C6 | 3:5:5:1 |
| M@HD@C6 | Macrophage membrane/ HA-TK-PEG-Chol/ DOTAP/DOPE/C6 | 14:3:5:5:1 |
| HD@DIR | HA-TK-PEG-Chol/ DOTAP/DOPE/DIR | 3:5:5:1 |
| M@HD@DIR | Macrophage membrane/ HA-TK-PEG-Chol/ DOTAP/DOPE/DIR | 14:3:5:5:1 |

**Fig. S1 Synthetic route of** **HA-TK-PEG-Chol**.

**Fig. S2 Spectra of HA-TK-PEG-Chol and its intermediates**. (A) ^1^H-NMR spectra of Chol-TK-COOH. (B) HRMS of Chol-TK-COOH. (C) ^1^H-NMR spectra of HA-TK-PEG-Chol. (D) ^1^H-NMR spectra of Chol-NH_2_, Chol-TK-COOH, HA, HA-TK-COOH, HA-TK-PEG-NH_2_ and HA-TK-PEG-Chol. (E) DOSY NMR spectra of HA-TK-PEG-Chol. (F) GPC spectra of HA-TK-PEG-Chol.

**Fig. S3** Expression and quantification of ADAM17 protein levels in HL-1 cells transfected with siADAM17-1 (A) and siADAM17-2 (B) (n = 4). The data are presented as the mean ± SD; Statistical differences were determined by t-test; ***p* < 0.01 versus the siNC group.

**Fig. S4** (A) Agarose gel electrophoresis at incremental DD/siADAM17 N/P ratios. (B) Agarose gel electrophoresis at incremental HCDD/ siADAM17 N/P ratios.

**Fig. S5** ζ potential diagram of siRNA, DD, DA, HC, HCDA and M@HCDA.

**Fig. S6** Agarose gel electrophoresis of siADAM17, HCDA and M@HCDA incubated with 10% FBS for different durationsat.

**Fig. S7** Images of M@HD@C6 and HD@C6 uptake by AC16 cells (A) and NRCFs (B) at different time periods (n = 6). The data are presented as the mean ± SD; Statistical differences were determined by one-way ANOVA; **p* ＜ 0.05, ***p* ＜ 0.01.

**Fig. S8** (A) Fluorescence intensity quantification of *ex vivo* hearts from different groups. (B) Fluorescence overlay images of various organs from M@HD@DIR, HD@DIR and DIR groups 6 h, 12 h, 24 h after injection of respective preparations. (n = 3). The data are presented as the mean ± SD; Statistical differences were determined by one-way ANOVA; **p* ＜ 0.05, ***p* ＜ 0.01.

**Fig. S9** Hemolysis picture of M@HCDA at different concentration (n = 3). The data are presented as the mean ± SD; Statistical differences were determined by one-way ANOVA; ***p* < 0.01 versus the Triton X-100 group.

**Fig. S10 Acute toxicity test of M@HCDA *in vivo***. (A) Schematic diagram of acute toxicity test procedure. (B, C) Electrocardiogram and cardiac ultrasound B-mode, M-mode, and PW Doppler images from each group of mice 24 hours after tail vein injection of different concentrations of M@HCDA or PBS. (D) The numerical values of EF%, FS%, LVID d, LVID s, LVEDV, LVESV and E/A Ratio in each group. (E) H&E staining images of the main metabolic organs of mice in each experimental group. (F) Expression levels of serum biochemical analysis indicators in mice of each experimental group. (n = 6). The data are presented as the mean ± SD; Statistical differences compared with the Vehicle group were determined by one-way ANOVA.

**Fig. S11** Analyses of heart weight/body weight (HW/BW) ratio (n = 6). The data are presented as the mean ± SD; Statistical differences compared with the Vehicle group were determined by one-way ANOVA.

**Fig. S12** Analyses of heart weight/body weight (HW/BW) ratio (n = 6). The data are presented as the mean ± SD; Statistical differences compared with the Vehicle group were determined by one-way ANOVA.

**Fig. S13** **Chronic toxicity test of M@HCDA *in vivo***. (A) Schematic diagram of chronic toxicity test procedure. (B, C) Electrocardiogram and cardiac ultrasound B-mode, M-mode, and PW Doppler images from each group of mice 4 weeks after tail vein injection of PBS or M@HCDA (400 μg/kg) first time. (D) The numerical values of EF%, FS%, LVID d, LVID s, LVEDV, LVESV and E/A Ratio in each group. (E) H&E staining images of the main metabolic organs of mice in each experimental group. (F) Expression levels of serum biochemical analysis indicators in mice of each experimental group. (n = 6). The data are presented as the mean ± SD; Statistical differences compared with the Vehicle group were determined by t-test.

**Fig. S14** Heatmap of the DEGs from DOX group versus Vehicle group (A) and DOX+M@HCDA group versus DOX group (B).

**Fig. S15** The FPKM of GPX4 and SLC7A11 in Vehicle, DOX and DOX+M@HCDA groups.

**Fig. S16** GSEA cnetplot of KEGG pathway enrichment analysis in DOX treated mice myocardial tissue versus Vehicle-treated mice.

**Fig. S17** GSEA cnetplot of KEGG pathway enrichment analysis in DOX and M@HCDA both treated mice myocardial tissue versus DOX-treated mice.

**Fig. S18** (A) Immunoblotting analysis of ADAM17 protein level and (B) ADAM17 mRNA level in the hearts (n = 3). The data are presented as the mean ± SD; Statistical differences were determined by one-way ANOVA; **p* ＜ 0.05, ***p* ＜ 0.01.

**Fig. S19** Immunoblotting analysis of GPX4 protein level in the HL-1 cells (up) and quantification (down, n = 3). The data are presented as the mean ± SD; Statistical differences were determined by one-way ANOVA; **p* ＜ 0.05, ***p* ＜ 0.01.

**Fig. S20** The levels of serum MDA in each group. The data are presented as the mean ± SD; Statistical differences were determined by one-way ANOVA; **p* ＜ 0.05, ***p* ＜ 0.01.
